# Evolutionary Relationships and Biogeography of the Ant-Epiphytic Genus *Squamellaria* (Rubiaceae: Psychotrieae) and Their Taxonomic Implications

**DOI:** 10.1371/journal.pone.0151317

**Published:** 2016-03-30

**Authors:** Guillaume Chomicki, Susanne S. Renner

**Affiliations:** Systematic Botany and Mycology, University of Munich (LMU), Menzinger Str. 67, 80638, Munich, Germany; Institute of Botany, CHINA

## Abstract

Ecological research on ant/plant symbioses in Fiji, combined with molecular phylogenetics, has brought to light four new species of *Squamellaria* in the subtribe Hydnophytinae of the Rubiaceae tribe Psychotrieae and revealed that four other species, previously in *Hydnophytum*, need to be transferred to *Squamellaria*. The diagnoses of the new species are based on morphological and DNA traits, with further insights from microCT scanning of flowers and leaf *δ*^*13*^*C* ratios (associated with Crassulacean acid metabolism). Our field and phylogenetic work results in a new circumscription of the genus *Squamellaria*, which now contains 12 species (to which we also provide a taxonomic key), not 3 as in the last revision. A clock-dated phylogeny and a model-testing biogeographic framework were used to infer the broader geographic history of rubiaceous ant plants in the Pacific, specifically the successive expansion of *Squamellaria* to Vanuatu, the Solomon Islands, and Fiji. The colonization of Vanuatu may have occurred from Fiji, when these islands were still in the same insular arc, while the colonization of the Solomon islands may have occurred after the separation of this island from the Fiji/Vanuatu arc. Some of these ant-housing epiphytes must have dispersed with their specialized ants, for instance attached to floating timber. Others acquired new ant symbionts on different islands.

## Introduction

The angiosperm family with the highest diversity of ant-plant is the Rubiaceae [1]. In Southeast Asia, it is the tribe Psychotrieae that is especially rich in epiphytic species occupied by ants living in specialized domatia. During ecological research on ant/plant symbioses in the Psychotrieae of Fiji, we discovered several new species that we are here placing in a phylogenetic and biogeographic context. Based on molecular-phylogenetic data, the four new species belong in the genus *Squamellaria* in the Hydnophytinae, a subtribe erected by Huxley and Jebb [2] to set apart a group of epiphytic ant plants from the rest of the Psychotrieae, which contain over 2000 species. The Hydnophytinae include about 100 species in five genera *Hydnophytum* (55 species; Jebb and Huxley, unpublished revision), *Myrmecodia* (26 species; [3]), *Myrmephytum* (5 species; [4]), *Anthorrhiza* (9 species; [5]), and *Squamellaria* (3 species; [6]). These five genera share a unique synapomorphy consisting of a hypocotyl-derived tuber (domatium) that contains a network of galleries, connected to the exterior by entrance holes. The galleries and entrance holes form regardless of the presence of ants [7, 8]. The tubers of most Hydnophytinae are inhabited by ants, usually belonging to the dolichoderine genera *Philidris* and *Anonychomyrma* [9]. In most species, the walls of the galleries inside the domatium are of two types: smooth walls (where the ants nest) and warted walls with small root-like protuberances [7,8,10]. Heim [11] suggested that the warts might be absorptive, a suggestion supported by Janzen [12], who on Borneo observed workers of *Philidris myrmecodiae* placing dead insects inside warted chambers, indicating to a trophic mutualism. The demonstration of such a mutualism came from a seminal paper by Huxley [9] who used radiolabelled sugar solutions to prove that molecules taken up by the ants moved from their feces in the warted cavities into the plants. Huxley [9] also provided evidence for an additional anti-herbivore defence role of the symbiotic ants.

Systematic work on the ant plant species in the Hydnophytinae began with the research of the Italian botanist Odoardo Beccari (1843–1920) who spent 13 years in Sarawak (1865–1878) and undertook two expeditions to West Papua, one in 1872, the other in 1875 [13]. He described numerous ant-housing species, notably in *Myrmecodia* W.Jack and *Hydnophytum* W.Jack [14]. Beccari also studied relevant herbarium material, including the first ant-plant ever collected on Fiji, a specimen prepared by members of the Wilkes United States Exploring Expedition in 1840 and described by Asa Gray as *M*. *imberbis* (Wilkes Expl. Exped. s.n.; US Catalog No.: 62266, barcode: 00129869). John Horne (1848–1928), a British forester living in Fiji in 1876/1877, collected a second ant-housing species, *H*. *wilsonii* [15], a name validated by Baker [16]. Based on the Wilkes and Horne specimens, Beccari thought that these Fijian ant plants differed sufficiently from *Hydnophytum* and *Myrmecodia* species to deserve a separate genus (a decision supported by DNA sequences; *Results*). He diagnosed the new genus by the presence of fringed scales (squamellae) at the inner base of the flower petals (Beccari [14], p. 228: “tubo intus ad basin squamulis 4 barbatis aucto”), and accordingly named it *Squamellaria*, with the new combinations, *S*. *imberbis* (A.Gray) Beccari and *S*. *wilsonii* (Horne ex Baker) Beccari. Beccari could not know whether the two species of *Squamellaria* formed the inflated hypocotyl tubers found in all species of this group (Hydnophytinae), writing “Gli esemplari di *M*. *imberbis* che conosco, constant soltanto di rami e mancano di radici o di tubero. Non trovo nemmeno alcuna citazione che mi faccia credere che queste due piante producano alla base un rigonfiamento abitato da formiche come gli *Hydnophytum*” (Beccari [14], p. 228), meaning “The specimens of *M*. *imberbis* that I have seen consist only of branches and lack roots or tubers. I also cannot find any observations that make me believe that these two plants [*M*. *imberbis* and *S*. *wilsonii*] produce a swelling at the base inhabited by ants, as do *Hydnophytum*.

On Fiji, cars came into use in the early 20^th^ century, and when A.C. Smith began collecting in the Fiji Archipelago in 1933–1934 [17], the road network was still limited, especially in the eastern part of Taveuni Island (Bouma), where roads were first built in the 1970’s [18]. With the increasingly easy access, a third species of *Squamellaria*, *S*. *major*, was discovered in 1953 by Smith [19] on the slopes of Mt. Manuka near Waikiri (*Smith 8323*; US Catalog No.: 2191043, barcode: 00129863), and a fourth, *S*. *thekii*, in 1983 by Jebb [6] from Taveuni at DesVoeux Peak near Somosomo (*Jebb 477*; BISH, K image barcode K000761985, SUVA). Benefitting from these known locations and easier road access, we conducted fieldwork in Viti Levu, Vanua Levu, and Taveuni, in 2014 and 2015, studied relevant herbarium material (see *Acknowledgments*), and used molecular phylogenetics to answer the following questions: (*i*) What are the species relationships in the Pacific Hydnophytinae and (*ii*) Are are the Pacific Hydnophytinae part of a single lineage that dispersed throughout the Pacific? We enhance our species descriptions with CT scanning of flowers and δ ^13^C to account for photosynthetic types (Table 1).

**Table 1:** δ ^13^C value in Fijian *Squamellaria*. A value below 20‰ is suggestive of CAM metabolism, around 20‰ implies an intermediate CAM/C3 metabolism and above 20‰ indicates C3 metabolism. In all cases, Isotope-Ratio Mass Spectrometry (IR-MS) measurements were measured on leaves.

Species	Voucher	δ ^13^C (‰)	Metabolism
***Squamellaria grayi sp*. *nov*.**	G. Chomicki, J. Aroles, A. Naikatini 53 (M)	-24.35	C3
***Squamellaria huxleyana sp*. *nov*.**	G. Chomicki, J. Aroles, A. Naikatini 48 (M)	-18.89	CAM
***Squamellaria imberbis***	G. Chomicki, J. Aroles, A. Naikatini 50 (M)	-28.01	C3
***Squamellaria jebbiana sp*. *nov*.**	G. Chomicki, J. Aroles, A. Naikatini 74 (M)	-29.79	C3
***Squamellaria major***	M.P.H. Jebb 475 (FHO)	-15.08	CAM
***Squamellaria major***	G. Chomicki, J. Aroles, A. Naikatini 61 (M)	-16.23	CAM
***Squamellaria tenuiflora* comb. Nov.**	G. Chomicki, J. Aroles, A. Naikatini 75 (M)	-34.72	C3
***Squamellaria tenuiflora* comb. Nov.**	G. Chomicki, J. Aroles, A. Naikatini 78 (M)	-34.01	C3
***Squamellaria thekii***	G. Chomicki, J. Aroles, A. Naikatini 57 (M)	-20.13	Intermediate
*Squamellaria wilkinsonii* comb. Nov.	G. Chomicki, J. Aroles, A. Naikatini 43 (M)	-30.44	C3
***Squamellaria wilkinsonii* comb. Nov.**	G. Chomicki, J. Aroles, A. Naikatini 45 (M)	-30.18	C3
***Squamellaria wilsonii***	G. Chomicki, J. Aroles, A. Naikatini 67 (M)	-30.19	C3

## Materials and Methods

### Ethics statement

Some of the new species reported in this work were collected in non-protected forests (*S*. *huxleyana*, along the cross-island road on Vanua Levu), others in protected forests (*S*. *grayi* in the Bouma heritage reserve on Taveuni and Waisali Forest Park on Vanua Levu; *S*. *jebbiana* DesVoeux peak reserve on Taveuni). All fieldwork was conducted jointly with members of the University of the South Pacific, Suva, Fiji (see *Acknowledgments*), thus no permits were required for the described study, which complied with all relevant regulations. The research did not endanger any protected species. Holotypes of our new species and duplicates of other collections are deposited in the SUVA herbarium in Fiji.

### Nomenclature

The electronic version of this article in Portable Document Format (PDF) in a work with an ISSN or ISBN will represent a published work according to the International Code of Nomenclature for algae, fungi, and plants, and hence the new names contained in the electronic publication of a PLOS article are effectively published under that Code from the electronic edition alone, so there is no longer any need to provide printed copies.

In addition, new names contained in this work have been submitted to IPNI, from where they will be made available to the Global Names Index. The IPNI LSIDs can be resolved and the associated information viewed through any standard web browser by appending the LSID contained in this publication to the prefix http://ipni.org/. The online version of this work is archived and available from the following digital repositories: PubMed Central and LOCKSS.

### DNA extraction, amplification, sequence alignment and phylogenetic inference

The isolation of DNA, amplification, and sequencing followed standard procedures, described in Chomicki and Renner [1, 20]. We sequenced and combined six plastid regions (*trnL* intron, *trnL*-*trnF* spacer, *ndhF*, *rps12*-*rpl20*, *trnS*-trnG, and *rps16*) and three nuclear regions (18S, ITS, and ETS) from 17 *Squamellaria* specimens. We sampled all species of Pacific Hydnophytinae (Fiji, Vanuatu, the Solomons). Vouchers, with their geographic origin and herbarium deposition, as well as the GenBank accession numbers for new sequences linked to this paper are shown in S1 Table. Dense species sampling of Hydnophytineae assured that the monophyly of *Squamellaria* could be rigidly tested. Sequence alignments were performed in MAFFT vs. 7 [21], under standard settings except for the ITS region aligned using the Q-INS-S option, which takes into consideration RNA secondary structure, as recommended for this marker. In the absence of statistically supported incongruence (defined as maximum likelihood bootstrap support >70%), we concatenated the datasets manually in Mesquite v. 2.75 [22]. Maximum likelihood tree inference relied on RAxML v. 8.1 [23], with 100 ML bootstrap replicates, using the GTR + G substitution model with six rate categories. We also conducted Bayesian analyses in MRBAYES v. 3.2 [24], using the best-fitting models identified by jModelTest2 [25] in a two-partition (chloroplast-nuclear) scheme. We used the default four chains (one cold and three heated), with uniform priors on most parameters. Substitution models for plastid (HYK+G) and nuclear (JC+G) were unlinked. The Markov chain Monte Carlo (MCMC) was run for 1 million generations, with parameters and trees sampled every 1,000 generations.

### Molecular clock dating

Molecular dating analyses relied on BEAST v. 2 [26] and uncorrelated lognormal relaxed clock models. We used the GTR + G substitution model with four rate categories and a Yule tree prior. The MCMCs were run for 20 million generations, with parameters and trees sampled every 10,000 generations. We used Tracer v. 1.6 [27] to check that the effective sample size (ESS) of all parameters was >200, indicating that runs had converged. After discarding 20% as burn-in, trees were summarized in TreeAnnotator v. 1.8 (part of the BEAST package) using the options ‘maximum clade credibility tree’, which is the tree with the highest product of the posterior probability of all its nodes, ‘mean node height,’ and a posterior probability limit of 0.98. The final tree was visualized in FigTree v. 1.4 [28]. To calibrate our tree, we constrained the age of the root, i.e., the split between the Pacific clade and the so-called *Psychotria* clade IV of Barrabé et al. [29], to 22 ± 7 Ma, based on the age of this node estimated by these authors, using a normal prior and a standard deviation of 4 corresponding to the 95% confidence interval of Barrabé et al. [29].

### Historical biogeography

We coded the geographic ranges of the Hydnophytinae and outgroup species as A = Fiji, B = Solomons, C = Vanuatu, D = Papua New Guinea, E = Australia, F = Wallis and Futuna, and G = Malesian region, H = Philippines, I = New Caledonia, J = Hawaii, K = French Polynesia. To infer ancestral areas, we used the multimodel approach implemented in the R package BioGeoBEARS [30, 31] and the chronogram obtained from the dating analysis in BEAST. BioGeoBEARS permits comparison of three biogeographic models, called dispersal-extinction-cladogenesis (DEC), dispersal-vicariance (DIVALIKE), and BAYAREA (BAYAREALIKE) [30, 31]. Founder-event speciation is modeled via a speciation parameter j that can be added to each of the models. We selected the best-fit model based on LogLikelihood values as well as the Akaike Information Criterion (ΔAICc). Statistics for these six models are shown in Table 2.

**Table 2:** Model-testing statistics from the BioGeoBEARS analysis. *d* refers to the rate of dispersal/range addition; *e*, to the extinction rate/range contraction; *j*, to the rate of founder-events. The best model (DEC+J) is highlighted in bold.

Models	LnL	Number of parameters	*d*	*e*	*j*
DEC	-117.86	2	0.0076	0.0284	0
**DEC+J**	**-86.44**	**3**	**1.00E-12**	**1.00E-12**	**0.0309**
DIVALIKE	-111.09	2	0.0073	0.0042	0
DIVALIKE+J	-86.95	3	1.00E-12	1.00E-12	0.0320
BAYAREALIKE	-133.49	2	0.0115	0.1052	0
BAYAREALIKE+J	-88.22	3	1.00E-07	1.00E-07	0.0309

### Measurement of δ^13^C values

We performed Isotope-Ratio Mass Spectometry (IR-MS) to detect possible *δ*^*13*^*C* differences among the species that might be associated with CAM versus C3 photosynthesis. We suspected such differences because *Squamellaria* plants are epiphytes growing in drought-stressed tree canopies. We collected and ground 1–2 mg samples of silica-dried leaves or stems of 12 specimens, representing all nine Fijian ant-plant species. Dried samples were analysed with a mass spectrometer at the geoscience institute of the University of Mainz, Germany. Results are reported in Table 1.

## Results

### Phylogenetic position of the four new species

**Fig 1:**
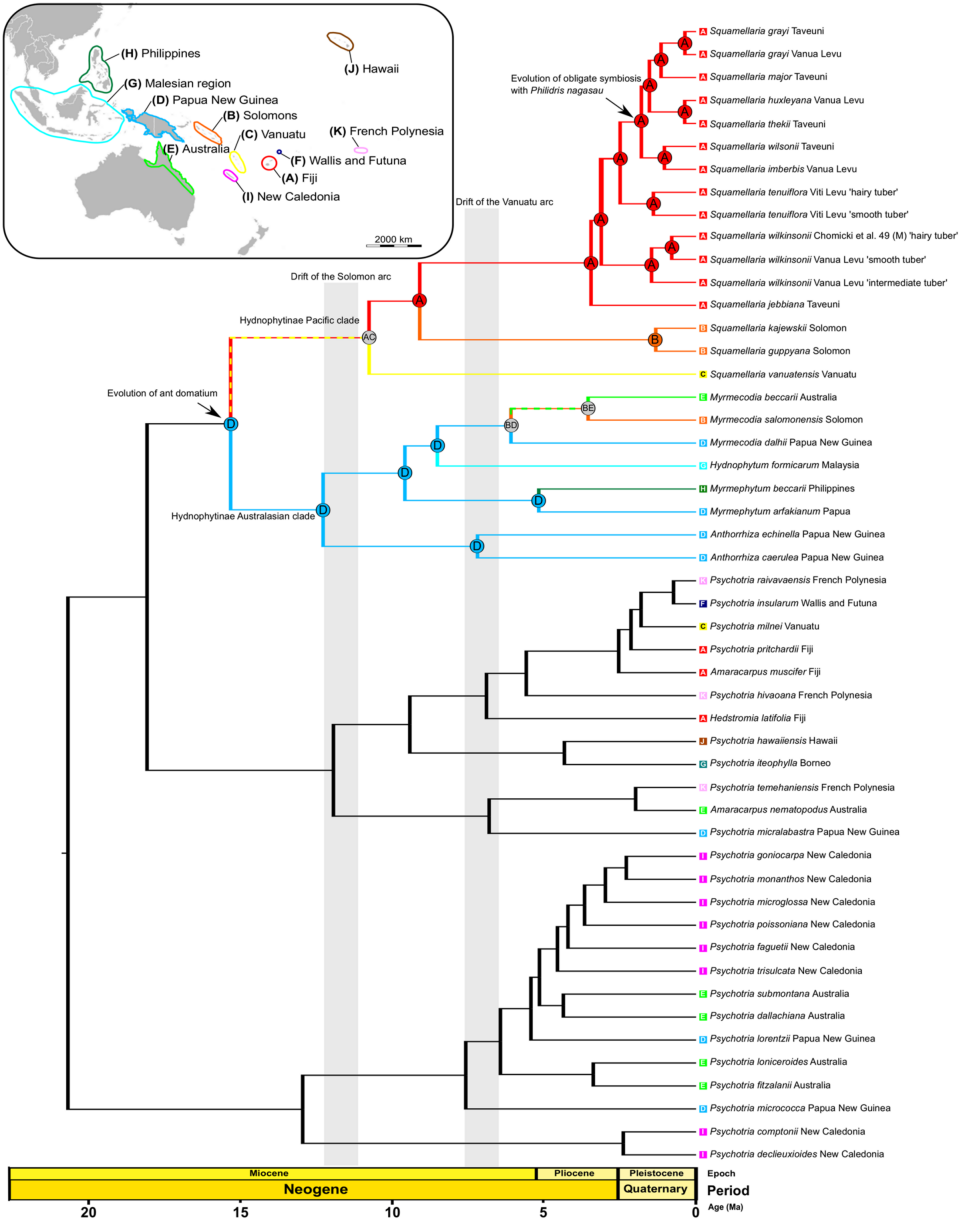
Phylogeny and biogeographic history of the Pacific Hydnophytinae, inferred under the DEC+J model (see Table 2) on the BEAST chronogram.

**Fig 2:**
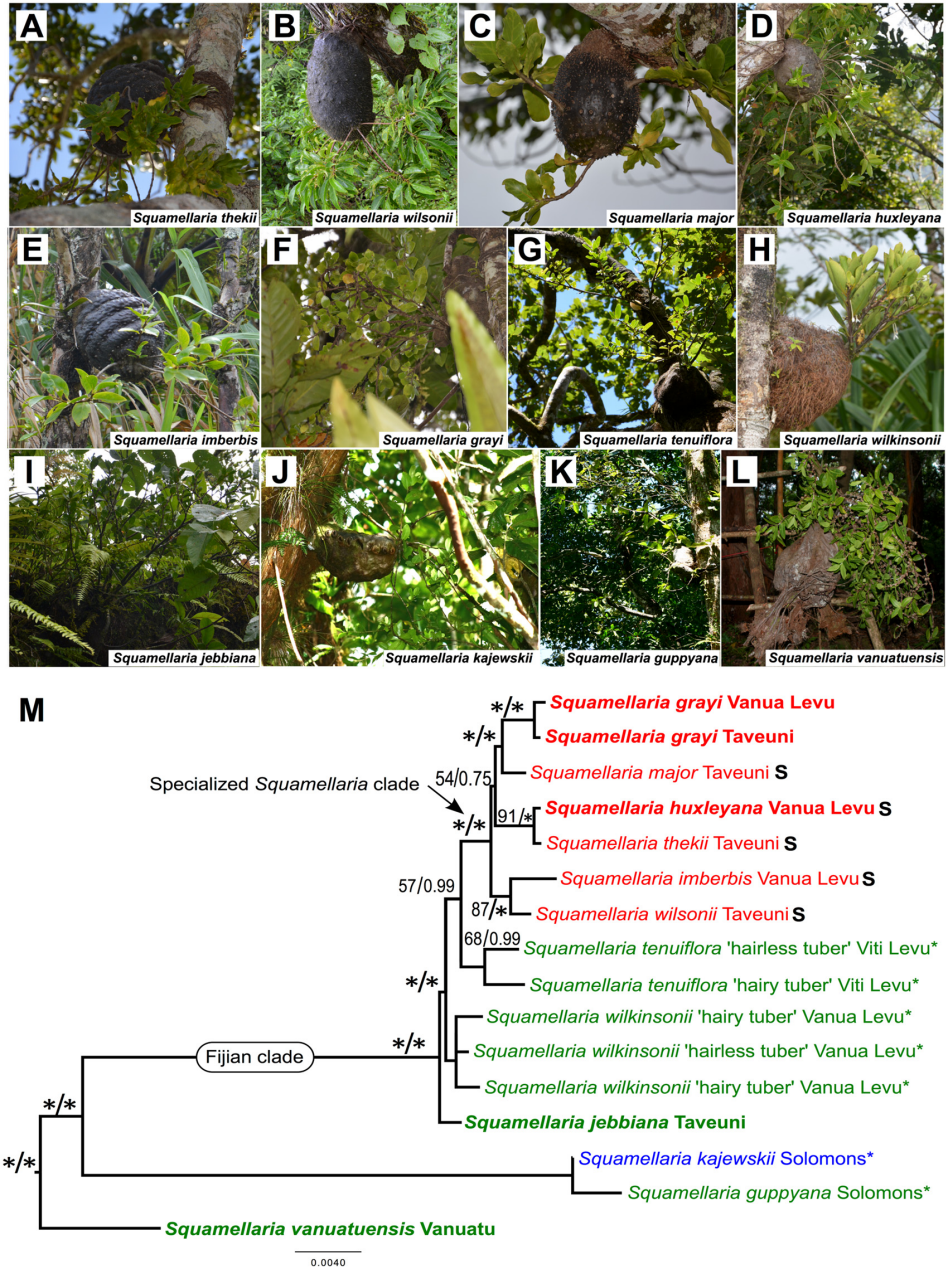
Photos of the 12 *Squamellaria* species and phylogenetic relationships among them. (**A**) *Squamellaria thekii*. (**B**) *S*. *wilsonii*. (**C**) *S*. *major*. (**D**) *S*. *huxleyana* Chomicki, sp. nov. (**E**) *S*. *imberbis*. (**F**) *S*. *grayi*, Chomicki & Wistuba, sp. nov. (**G**) *S*. *tenuiflora* comb. nov. (**H**) *S*. *wilkinsonii* comb. nov. (**I**) *S*. *jebbiana* Chomicki, sp. nov. (**J**) *S*. *kajewskii* comb. nov. (**K**) *S*. *guppyana* comb. nov. (**L**) *S*. *vanuatuensis*, sp. nov. (**M**) Maximum likelihood phylogenetic tree of the genus *Squamellaria* based on up to combined plastid and nuclear DNA regions (outgroups not shown). Numbers above branches are the maximum likelihood bootstrap support values, followed by the posterior probabilities from a Bayesian analysis of the same dataset. Asterisks above branches (*) indicate a maximal support (100 and 1 for ML and Bayesian analyses, respectively). Color-coding of the species names refers to obligate symbiosis with *Philidris nagasau* ants (red), facultative symbiosis with various ant species (green), or no symbiosis with ants (blue). Asterisks after species names refer to names that have been transferred to *Squamellaria*. An ‘S’ after species name refers to the presence of squamellae. Photographic credits: G. Chomicki except (J-K): Derrick Rowe and (L): Bruno Corbara.

Our maximum likelihood and Bayesian tree searches based on up to 9300 aligned nucleotides from the combined plastid and nuclear markers (S1 Table) support the monophyly of a group of species close to *S*. *imberbis*, the type species of *Squamellaria*, while the type species of *Hydnophytum*, *H*. *formicarum* Jack, is embedded in an Australasian clade of Hydnophytinae (Fig 1), supporting Beccari’s [14] gut feeling that the Fijian ant plant species are only distantly related to core-*Hydnophytum*. Four species of *Hydnophytum*, however, are more closely related to the type species of *Squamellaria* than that of *Hydnophytum* (marked with an asterisk in Fig 2M) and here transferred into *Squamellaria*. One of our new species, *S*. *grayi*, is placed as sister to *S*. *major*, the other, *S*. *huxleyana*, as sister to *S*. *thekii*, the third *S*. *jebbiana*, as sister to the remaining nine Fijian *Squamellaria* species, and the fourth, *S*. *vanuatuensis*, as sister to all other species in the genus (Figs 1 and 2M).

### Dated phylogeny and historical biogeography of Pacific ant-plants

The BioGeoBEARS analysis selected the model ‘Dispersal-Extinction-Cladogenesis + founder event speciation’ (DEC + J) as best explaining our data (Table 2), and our phylogenetic analysis revealed two main clades of Hydnophytinae (Fig 1): A Pacific clade consisting of *Squamellaria* and the four species previously in *Hydnophytum* and an Australasian clade consisting of *Anthorrhiza*, *Hydnophytum* (as to its type species), *Myrmecodia* and *Myrmephytum*. The Solomon Islands (color-coded orange) were colonized at least twice by epiphytic ant-plants, while Fiji and Vanuatu each were each colonized only once. The Pacific ant-plant clade apparently dates to 10.7 ± 5 Ma, when *S*. *vanuatuensis* diverged from the ancestor of the remaining species. We reconstructed the most recent common ancestor of *Squamellaria* as living in Fiji and Vanuatu (Fig 1), at a time when these two archipelagos were part of the same volcanic arc [32]. Colonization of the Solomons at 9.1 ± 4 Ma, led to the pair of Solomon endemics *S*. *kajewskii* and *S*. *guppyana*, which diverged from each other at 1.3 ± 1 Ma. The most recent common ancestor of the Fijian *Squamellaria* species (*S*. *imberbis*, *S*. *wilsonii*, *S*. *huxleyana*, *S*. *grayi* and *S*. *thekii*) inhabited by the ant species *Philidris nagasau* Mann (1921) is dated to 1.8 ± 1 Ma (Fig 1).

### Taxonomic treatment

**Fig 3:**
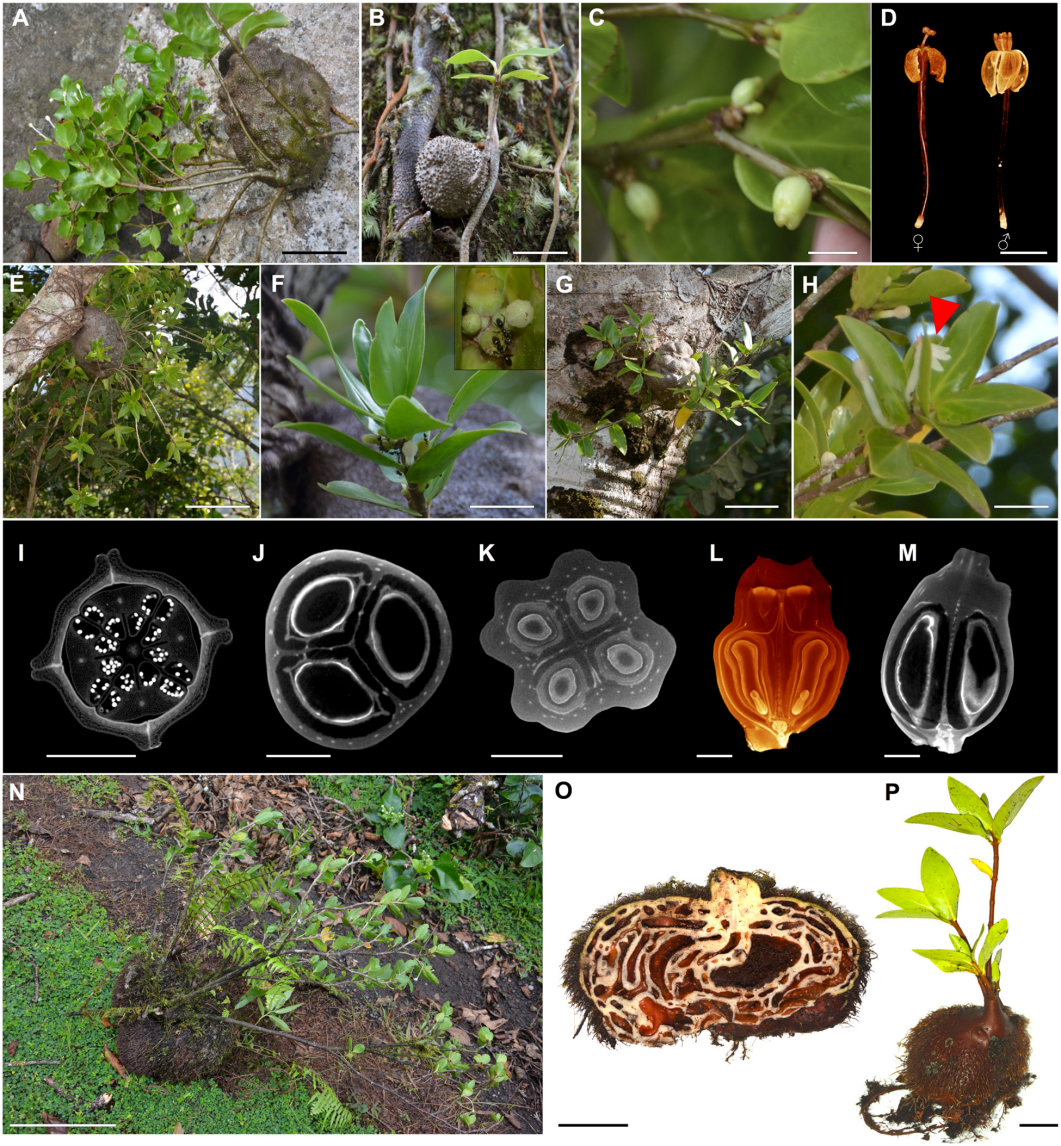
Photos of the three Fijian new species, *Squamellaria grayi*, Chomicki & Wistuba, sp. nov. (Taveuni), *S*. *huxleyana* Chomicki, sp. nov. (Vanua Levu) and *S*. *jebbiana* Chomicki, sp. nov. (**A-D)** *Squamellaria grayi*. (**A**) Mature adult with flowers closed during the day. (**B**) Seedling. (**C**) Fruits. (**D**) CT scanning image of the functionally unisexual flowers of *S*. *grayi* lacking the squamellae at the inner base of the flower tube (see also Fig 4B, 4E and 4F). (**E-H**) *Squamellaria huxleyana*. (**E**) Habit of a mature adult. (**F**) Shoot with calyx nectaries visited by *Philidris nagasau* workers. Inset shows details of nectary and fruits (see also Fig 6E). (**G**) Habit of two young individuals growing adjacently. (**H**) Flowering shoot including one flower whose corolla has split and which is therefore secondarily zygomorphic. (**I**) CT-scanning optical cross-section of *S*. *grayi* bud, with reduplicate petal margins (see also Fig 4C). (**J**) CT-scanning optical cross-section of *S*. *grayi* fruit, with three carpels. (**K**) CT-scanning cross-section of *S*. *huxleyana* bud, showing the four carpels. (**L**) CT-scanning longitudinal 3D reconstruction of an *S*. *huxleyana* fruit showing the curved pyrenes. (**M**) CT-scanning longitudinal section of *S*. *grayi* fruit showing the straight pyrenes. (**N-P**) *S*. *jebbiana*. (**N**) Habit of a mature adult (fall on the ground). (**M**) Domatium cross-section. (**O**) Juvenile individual. Photographic credit: G. Chomicki except D, I-M: Y. Staedler. Scale bars: A: 10 cm; B: 1.5 cm; C-D: 1 cm; E: 20 cm; F: 2 cm; G: 7 cm; H: 2 cm; I,J: 1.5 mm; K: 3 mm; L-M: 2 mm; N: 20 cm; O: 6 cm; P: 2.5 cm.

**Fig 4:**
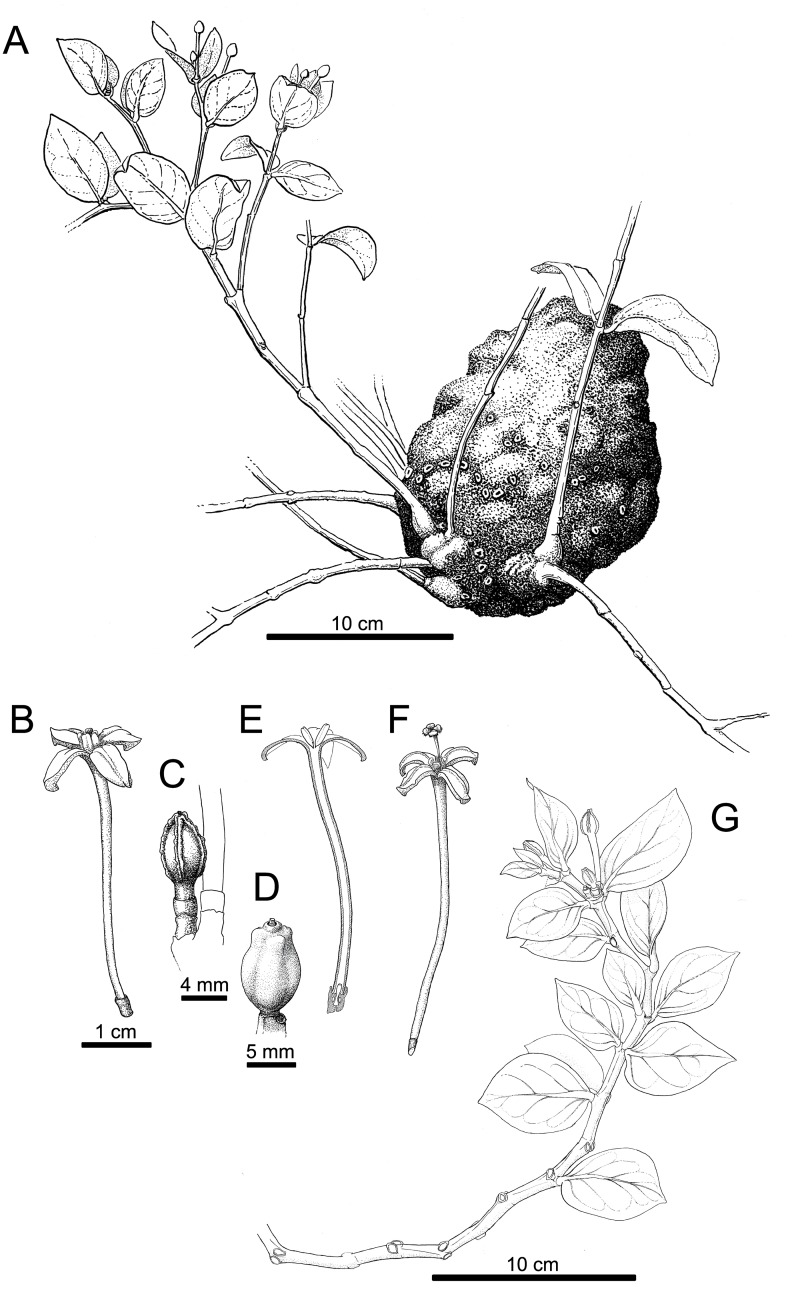
*Squamellaria grayi* Chomicki & Wistuba, spec. nov. (**A**) Habit showing the domatium. (**B**) Male flower. (**C**) Bud with reduplicate petal margins. (**D**) Fruit. (**E**) Male flower in longitudinal section. (**F**) Female flower. (**G**) Flowering shoot with anisophyllous paired leaves.

**Fig 5:**
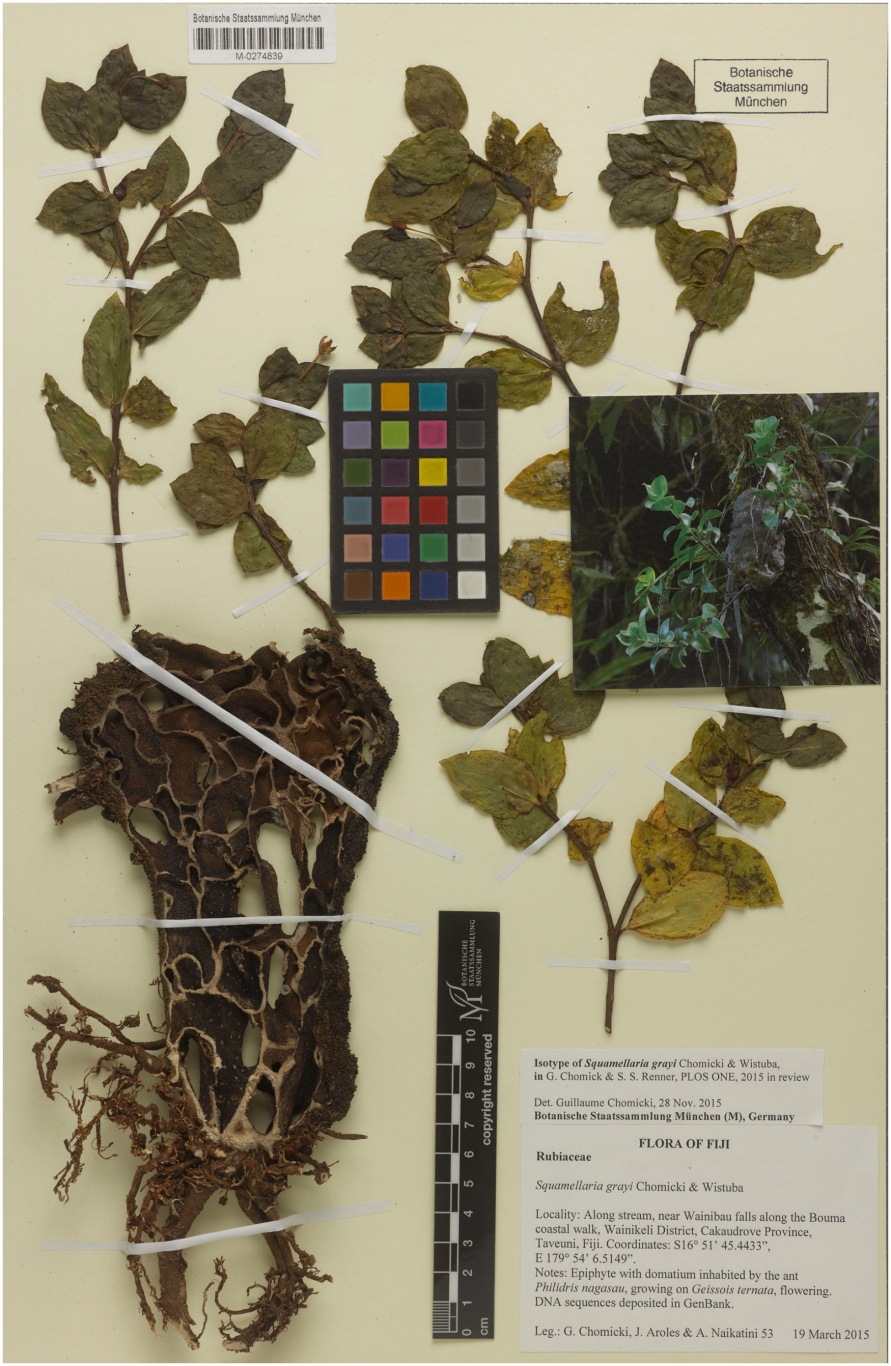
Isotype of *Squamellaria grayi*, G. Chomicki, J. Aroles, N. Naikatini 53 (M, barcode: M-0274839).

***Squamellaria grayi*** Chomicki & Wistuba spec. nov. [urn:lsid:ipni.org:names:77153474–1] (Figs 2F, 3A–3D, 4 and 5).

#### Type

FIJI. Taveuni: Lavena, at the end of Lavena coastal walk, ~4 km (by walk) NW of Lavena village, 16°49’58.98”S, 179°58’36.9”E, 19 m alt., 21 March 2015, *G*. *Chomicki*, *J*. *Aroles & A*. *Naikatini 53* (SUVA holotype; GH, K, L, M barcode: M-0274839, Fig 5, MO, NOU, NSW, P, S isotypes).

#### Diagnosis

*Squamellaria grayi* differs from all other species in the genus in its calyx length (2–3 mm vs. 5–7 mm in the other species), corolla tube width (2.5–3.5 mm vs. 5–8 mm), and three carpels with straight pyrenes (vs. four carpels and more or less curved pyrenes). It also differs in one substitution at position 372 (GenBank # KU586339) in the nuclear ribosomal intergenic spacer region ITS (C vs. A in all other *Squamellaria* species).

#### Description

Tuber (hypocotyle-derived ant-housing structure) attached to tree trunks (see Figs 2F and 3A and 3B), 15–25 to 25–40 cm, cylindrical, tuber apex flattened, surface with small, dark spiny protuberances (1–3 mm long), tuber surface grey, domatium entrance holes 1.5 to 3 mm wide, except the first one, which is 5–8 mm wide. Stems several, branched, of sympodial structure, alternating with entrance hole rings, often imperfect. Internodes 1–5 cm long and 0.2–1 cm in diameter, nodes slightly swollen (Fig 3A), 0.3–1 cm in diameter. Leaves arranged in a decussate phyllotaxis, slightly succulent but performing C3 photosynthesis, curved on the apical-basal axis (Fig 3A), slightly anisophyllous (Fig 4G), lamina ovate (Figs 3A and 4A and 4G), at each mature leaf pair, larger leaf 4–5 cm long, 2.5–3.5 wide and smaller leaf 2.5–4 cm long, 2–3 cm wide, leaves 2–3.5 mm thick, apex acute, base rounded, pale green on both the adaxial (upper) and abaxial (lower) sides, the petiole 2–5 mm long and 2–3.5 mm wide. Primary leaf vein monopodial, secondary veins brochidromous (i.e., secondary veins connect to the connects directly to the next secondary vein via a loop; Fig 4G). Inflorescences (and infructescences) on lateral short shoots, axillary and terminal (Figs 3A and 4G). Flowers functionally unisexual, plants monoecious, female flowers with four sterile stamens, male flowers with a sterile gynoecium (Figs 3D and 4B, 4E and 4F), actinomorphic, 3–5 cm long, opening mostly at night, strongly fragrant, opening only once. Calyx light green, with 4 fused sepals, to 3 mm long and 2 mm wide; corolla with 4 petals, white, glabrous, each petal distal lobe 0.8–1.3 cm long and 0.5–0.8 cm wide, whorled, in a valvate aestivation with revolute margins, corolla tube 2.5–4 cm long. Squamellae absent (Fig 4E). Anthers basifixed, adnate to corolla, valvate, and with introrse dehiscence. Ovary inferior, with three congenitally fused carpels. Stigma flattened, four-parted, square in section, slightly hairy (Fig 4F). Pyrenes 3, straight (Fig 3M). Fruit oblong, round in section, to 1 cm long and 7 mm wide (Fig 3M).

#### Floral formulae

Male: *K(4)[C(4)A(4)]G(3); Female: *K(4)[C(4)A^0^(4)]G(3)

#### Distribution and ecology

**Fig 6:**
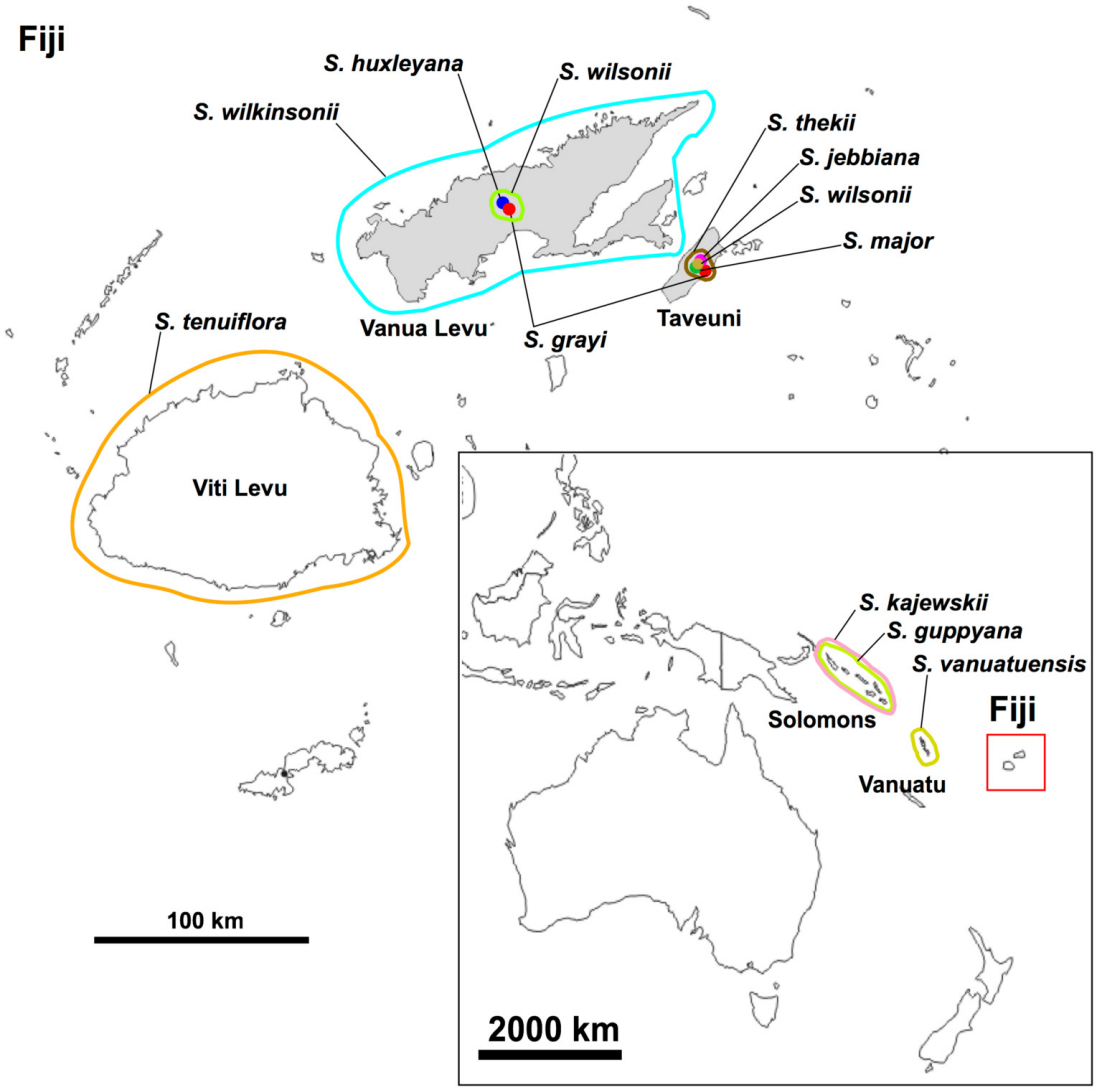
Geographic distribution of the 12 *Squamellaria* species.

*Squamellaria grayi* is known from the vicinity of Lavena on Taveuni (Fig 6), where it grows in forest at sea level, and from Waisali forest reserve in Central Vanua Levu, where it grows at low elevations. The species is inhabited by the Dolichoderinae ant species *Philidris nagasau*. At night, its flowers emit a strong sweet perfume while the other *Squamellaria* species from the clade obligately inhabited by *Philidris nagasau* (see Figs 1 and 2) flower during the day and lack any obvious sent. Its lack of scales (squamellae) at the inner petals bases (Fig 4E) appears to be a secondary loss (Fig 2M).

#### Etymology and common name

Named in honour of the American botanist Asa Gray who described the first *Squamellaria* (Gray [33]; cf. *Introduction*). Because the epithet *grayana* is occupied by *Psychotria grayana* K.Schum., we opted for *grayi*, so as avoid homonymy should *Squamellaria* be sunk into *Psychotria*. Like the other Fijian *Squamellaria*, *S*. *grayi* is locally called ‘theke theke nkau’ meaning testicles of the trees in Fijian, ‘theketuwawa’ meaning giant scrotum, or ‘theketheke’ meaning scrotum. Other names exist, but their meaning is uncertain ‘mokamoka’, ‘ndatokaikai’ (Alivereti Naikatini, pers. comm. to GC in June 2015).

#### Conservation status

The species is known from Lavena, where its range may be <10 km^2^, and from Waisali forest reserve on Vanua Levu. Fijian law protects plants growing in Bouma National Heritage Park and Waisali forest reserve. Although the lack of data prevents us from assigning an IUCN status to this species, we suspect that it is at least endangered based on criteria B (extent of occurrence) and C (population size and decline) [34].

#### Specimens examined

FIJI. Vanua Levu: Waisali forest park reserve, 16°38’19.8”S, 179°13’19.7”E, 219 m lat., 20 March 2015, *G*. *Chomicki*, *J*. *Aroles & A*. *Naikatini 47* (SUVA, GH, K, L, M, MO, NOU, NSW, P, S).

**Fig 7:**
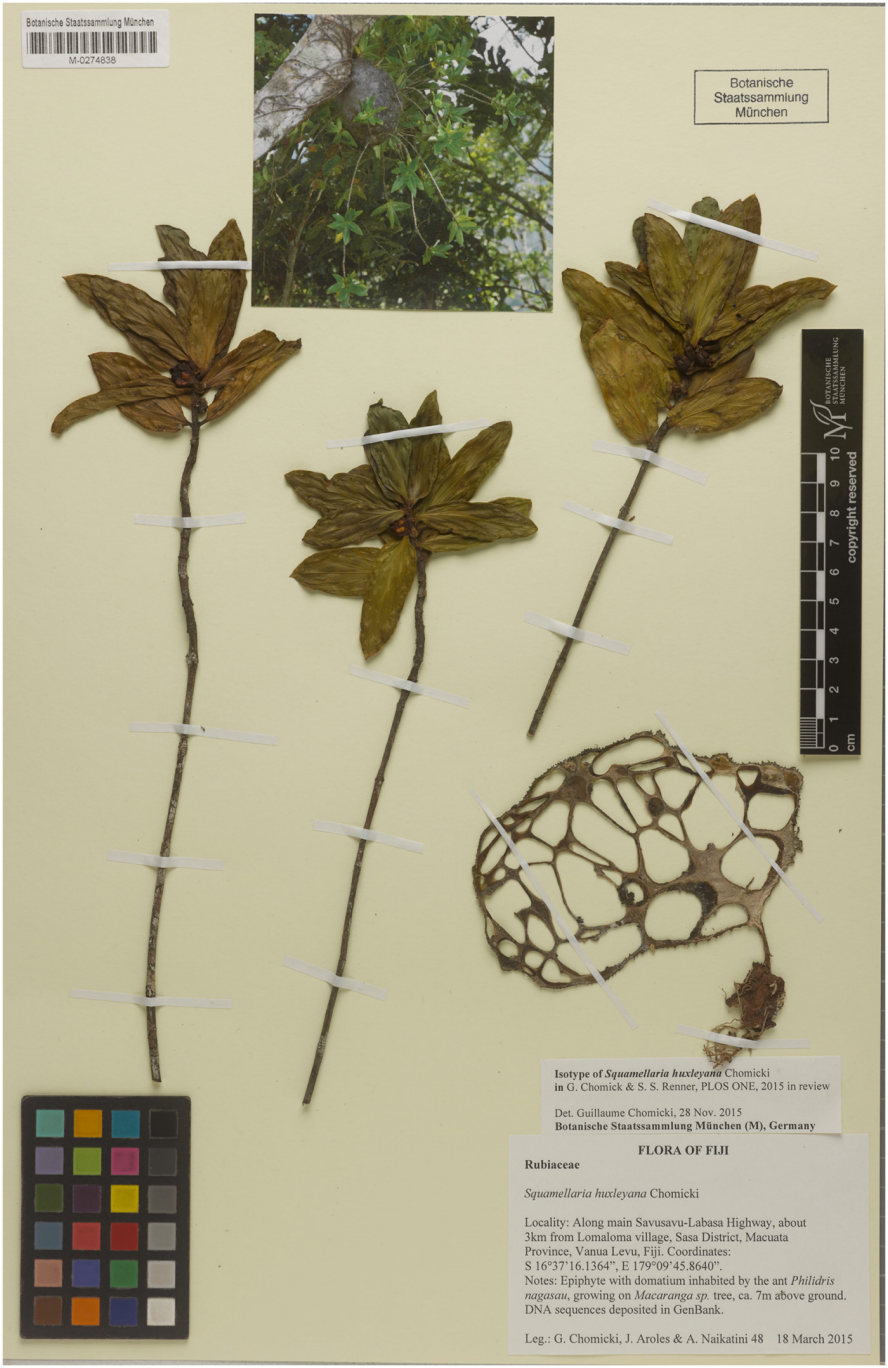
Isotype of *Squamellaria huxleyana*, G. Chomicki, J. Aroles, N. Naikatini 48 (M, barcode: M-0274838).

**Fig 8:**
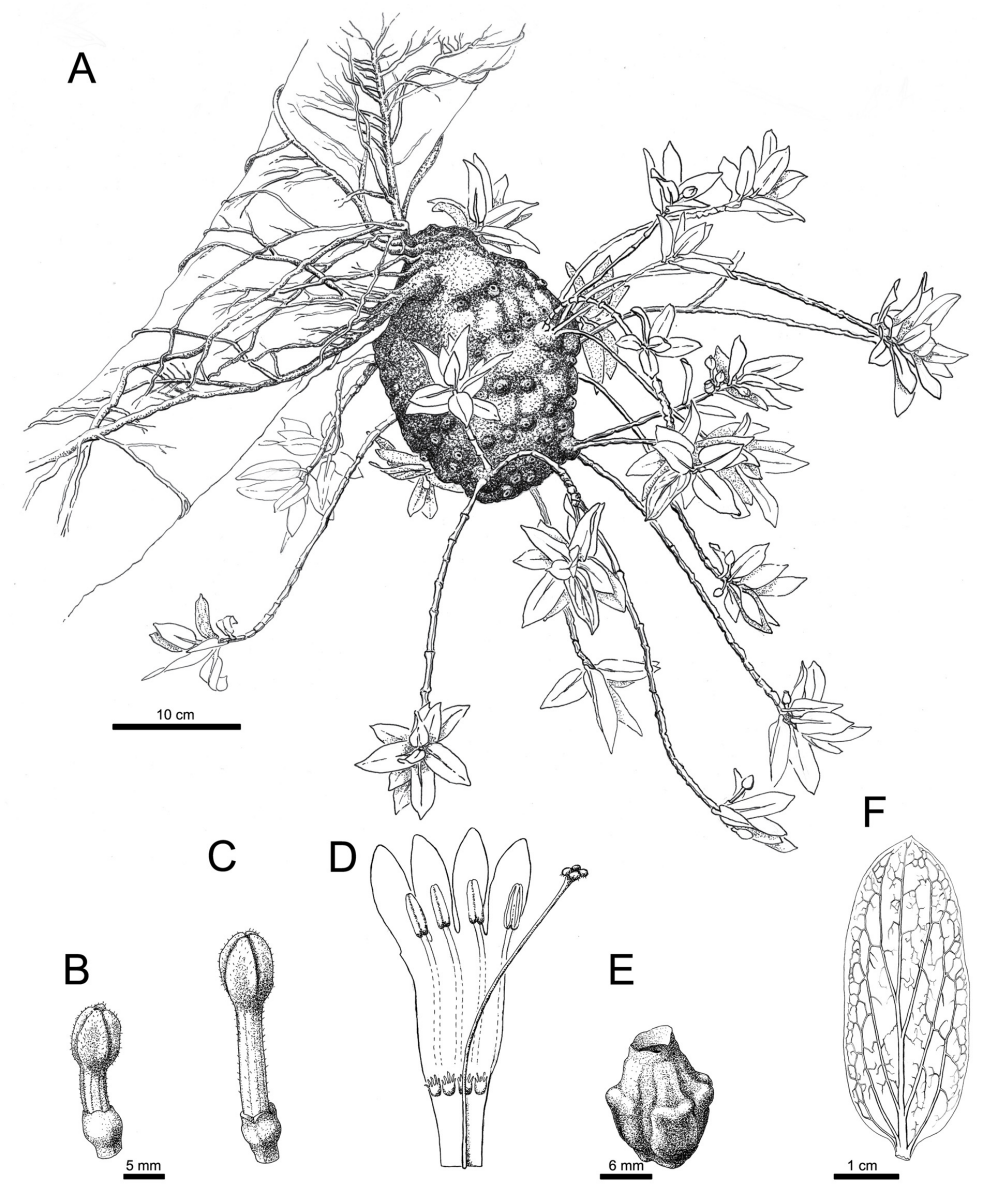
*Squamellaria huxleyana* Chomicki, spec. nov. (**A**) Habit showing the domatium. (**B**, **C**) Successive stages of flower bud opening, showing the corolla tube elongation. (**D**) Open corolla tube with the four squamellae. (**E**) Fruit with a bilobed-quadrangular shape in cross section. (**F**) Leaf, showing the festooned brochidromous venation (connecting to other secondary veins via multiple loops and not reaching the margin).

***Squamellaria huxleyana*** Chomicki **spec. nov.** [urn:lsid:ipni.org:names:77153475–1] (Figs 2D, 3E–3H, 3K and 3L, 7 and 8)

#### Type

FIJI. Vanua Levu: 45 km North of Savusavu towards Labasa, 16°37’16.1364”S, 179°09’45.864”E, 271 m alt., 18 March 2015, *G*. *Chomicki*, *J*. *Aroles & A*. *Naikatini 48* (SUVA holotype; GH, K, L, M barcode: M-0274838, Fig 7, MO, NOU, NSW, P, S isotypes).

#### Diagnosis

*Squamellaria huxleyana* differs from all other species in the genus by the following combination of characters: oblong leaves with a rounded base, 4–5 cm long, 1.5–2 wide, ant houses (domatia) regularly globose and grey with dark protuberances, and fruits bilobed-quadrangular in section (versus round or quandrangular, but not bilobed fruits in the remaining species).

#### Description

Tuber attached to tree trunks (see Figs 2D and 3E) 15–25 x 25–30 cm, globose (in juveniles, Fig 3G) to ovoid (in mature plants, Figs 1G and 4A), its apex convex, surface grey with small dark protuberances (1–3 mm long) (Figs 2D and 3E), ant entrance holes to 3 mm in diameter, except the first (oldest) one, which is 5–8 mm in diameter. Stems several, in clusters, alternate with entrance hole rings around the tuber (Figs 3E and 8A), rarely branched, of sympodial structure. Stem diameter larger at distal ends (~0.8 cm), which are fleshy, than at the base (~0.3–0.4 cm). Internodes 0.5–4 cm long, 0.3–0.8 cm in diameter, nodes slightly swollen (Figs 3F and 8A), 0.4–1 cm in diameter. Internode length decreasing towards the distal end of each shoot. Leaves decussate (Figs 3E–3F and 8A), lamina oblong, 4–5 cm long, 1.5–2 cm wide, 2–3.5 mm thick, performing CAM or intermediate C3/CAM photosynthesis, pale green with translucent, entire margins, apex acute, base rounded, petiole 2–5 mm long and 2–3.5 mm wide (Figs 3F and 8F). Primary leaf vein monopodial, secondary veins festooned brochidromous (connecting to other secondary veins via multiple loops and not reaching the margin; Fig 8F). Inflorescences consisting of lateral short shoots in terminal and axillary position. Flowers bisexual, homostylous, 3–4 cm long, after the first days of anthesis, initially actinomorphic but the corolla often splitting at full anthesis resulting in a secondary zygomorphy (monosymmetry) (Fig 3H). Calyx light green, cup-shaped to 5 mm long and 5 mm wide, with a large interior nectary gland (Fig 3L); corolla white, glabrous, 3–5 cm long, 4 petals, hairy on the outside, whorled, in a valvate aestivation, tube 2.5–3.5 cm, lobes ca. 3 x 5 mm. Squamellae (scales inside petals) present, 4, one at the base of each petal (Fig 8D). Anthers basifixed, valvate, with introrse deshiscence (Fig 8D). Ovary inferior, with four carpels congenitally fused. Stigma flattened, four-parted, square in section. Fruit turbinate at the base, bilobed-quandrangular in section ca. 1 cm long and 7 mm large (Figs 3K–3L and 8E). Pyrenes, 4, curved (Fig 3K–3L).

#### Floral formula

*(↓)K(4)[C(4)A(4)]G(4)

#### Distribution and ecology

*Squamellaria huxleyana* is only known from Vanua Levu, about 45 km northwest of Savusavu, along the road towards Labasa (Fig 6). It has been observed at elevations of 400–500 m in open areas and on trees. The species lives in obligate symbiosis with *Philidris nagasau* and is morphologically close to its sister species *S*. *thekii*, which occurs on Taveuni, but differs by the trait combination mentioned in the diagnosis. These two species appear to have evolved by allopatric speciation following dispersal between Taveuni and Vanua Levu, two islands only 6.5 km apart.

#### Etymology and common name

Named in honour of Camilla R. Huxley-Lambrick, née Huxley, for her key contributions to the biology and taxonomy of ant plants in the Rubiaceae [2–5, 9]. Its common names are the same as those of *S*. *grayi*.

#### Conservation status

The species is known from two localities separated by about one kilometre, and its range is likely <10 km^2^. We have not found *S*. *huxleyana* in Waisali forest reserve, and the two locations where we found it are not protected sites. Although the lack of data prevents us from assigning an IUCN status to this species, we suspect that it is at least endangered based on criteria B (extent of occurrence) and C (population size and decline) [34].

#### Specimens examined

Only known from the type collection.

**Fig 9:**
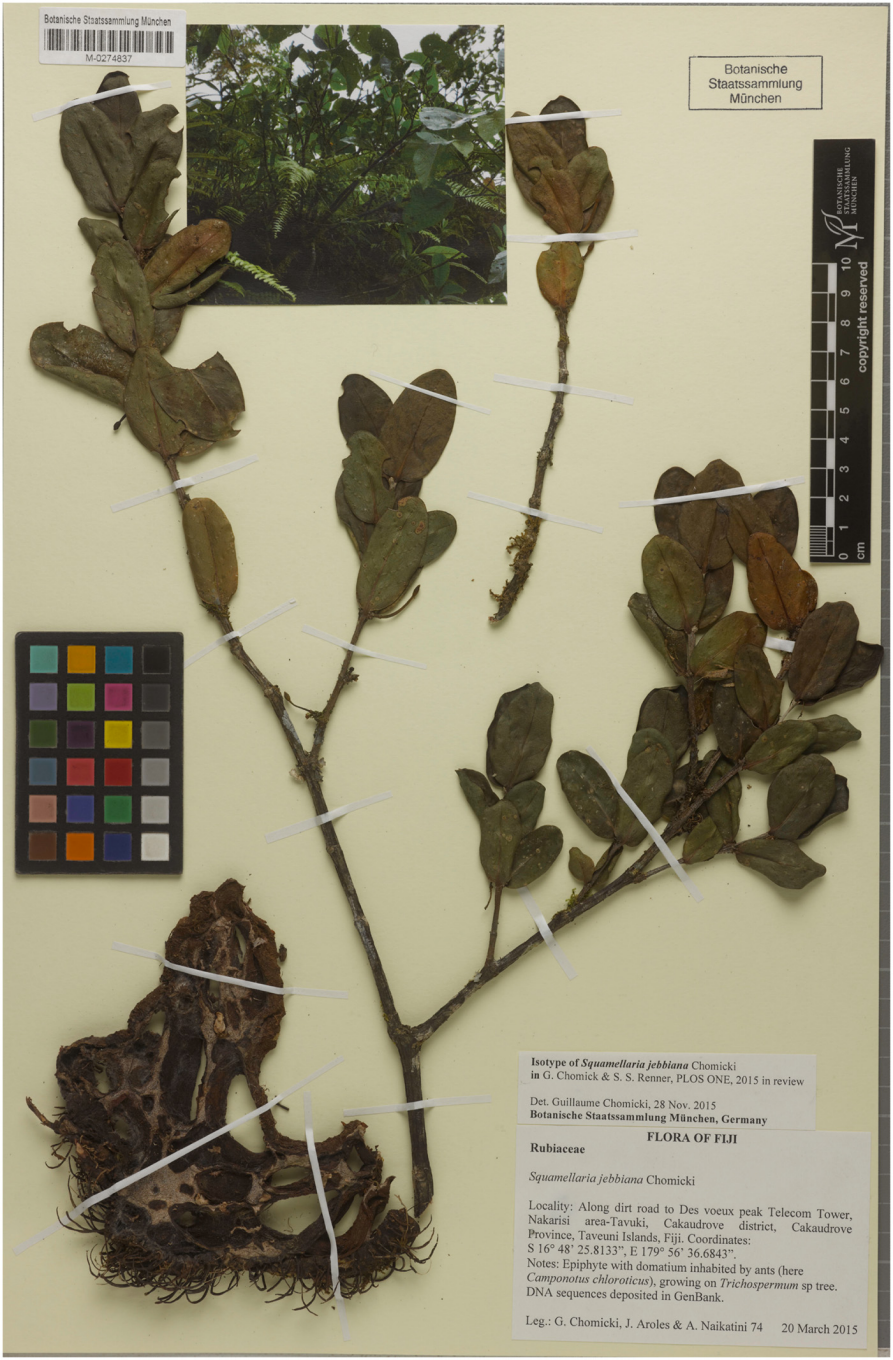
Isotype of *Squamellaria jebbiana*, G. Chomicki, J. Aroles, N. Naikatini 74 (M, barcode: M-0274837).

***Squamellaria jebbiana*** Chomicki **spec. nov.** [urn:lsid:ipni.org:names:77153477–1] (Figs 2I, 3N–3P and 9)

#### Type

FIJI. Taveuni: Des Voeux peak, 16°48’25. 8133”S, 179°56’36.6843”E, 450 m alt., 22 March 2015, *G*. *Chomicki*, *J*. *Aroles & A*. *Naikatini 74* (SUVA holotype; GH, K, L, M isotype barcode: M-0274837, MO, NOU, NSW, P, S isotypes).

#### Diagnosis

*Squamellaria jebbiana* differs from the remaining species by consistently elliptic leaves. Two substitutions can be used to diagnose this species: a C in position 354 of ITS (GenBank # KU586342) instead of an A or T, a C at position 278 of rps16 (GenBank # KU586438) instead of an A in all other Fijian *Squamellaria*.

#### Description

Tuber attached to tree trunks, 25–40 to 25–40 cm, conical, tuber apex prominent, its surface dark brown, domatium entrance holes of two kinds, lipped to 1.5 cm in diameter, and funnel-like, often ovate, to 6 cm in diameter. Stems several, branched, of sympodial structure (Fig 3N), all emerging from the prominent tuber apex (Fig 3N and 3O). Internodes 1–8 cm long and 0.3–1.5 cm in diameter, nodes slightly swollen (Fig 3N). Leaves decussate, lamina elliptic (Figs 3N and 9) but acuminate apex in juveniles (Fig 3P), leaf 3–5 cm long, 2.5–3.5 wide, 2–3.5 mm thick, slightly succulent but performing C3 photosynthesis, apex rounded to slightly convex, base rounded, darker green on the adaxial (upper) than on the abaxial (lower) sides, the petiole 2–5 mm long and 3–3.5 mm wide. Primary leaf vein monopodial, secondary veins brochidromous (i.e., secondary veins connect to the connects directly to the next secondary vein via a loop; Fig 9). Inflorescences (and infructescences) on lateral short shoots, axillary and terminal (Figs 3N and 9). Flowers functionally unisexual, plants monoecious female flowers with four sterile stamens, male flowers with a sterile gynoecium (Figs 3N and 9), actinomorphic, 3–5 cm long. Calyx light green, made of 4 fused sepals, to 2 mm long and 2 mm wide; corolla with 4 petals, white, glabrous, whorled, in a valvate aestivation with revolute margins, corolla tube 2.5–4 cm long. Anthers basifixed, adnate to corolla, valvate, and with introrse dehiscence. Ovary inferior, flattened, with two congenitally fused carpels. Pyrenes 2, straight. Fruit oblong, ovate in section, to 1 cm long and 7 mm wide.

#### Floral formulae

Male: *K(4)[C(4)A(4)]G(2); Female: *K(4)[C(4)A^0^(4)]G(2)

#### Distribution and ecology

*Squamellaria jebbiana* is known from the path going from Somosomo to DesVoeux peak and Mt. Manuca, where it grows from 400 m to 600 m. It is usually inhabited by *Camponotus* ants (*C*. *chloroticus*) and several species of *Pheidole*, but not the Dolichoderinae *Philidris nagasau*.

#### Etymology and common name

Named in honour of the Irish botanist Matthew P. H. Jebb for his contributions to the taxonomy and biology of the Hydnophytinae [2–6, 35]. Its common names are the same as those of *S*. *grayi* and *S*. *huxleyana*.

#### Conservation status

The species is known from the DesVoeux peak reserve, where its range may be <10 km^2^. Fijian law protects plants growing in DesVoeux peak reserve. Although the lack of data prevents us from assigning an IUCN status to this species, we suspect that it is at least endangered based on criteria B (extent of occurrence) and C (population size and decline) [34].

#### Specimens examined

Only known from the type collection.

**Fig 10:**
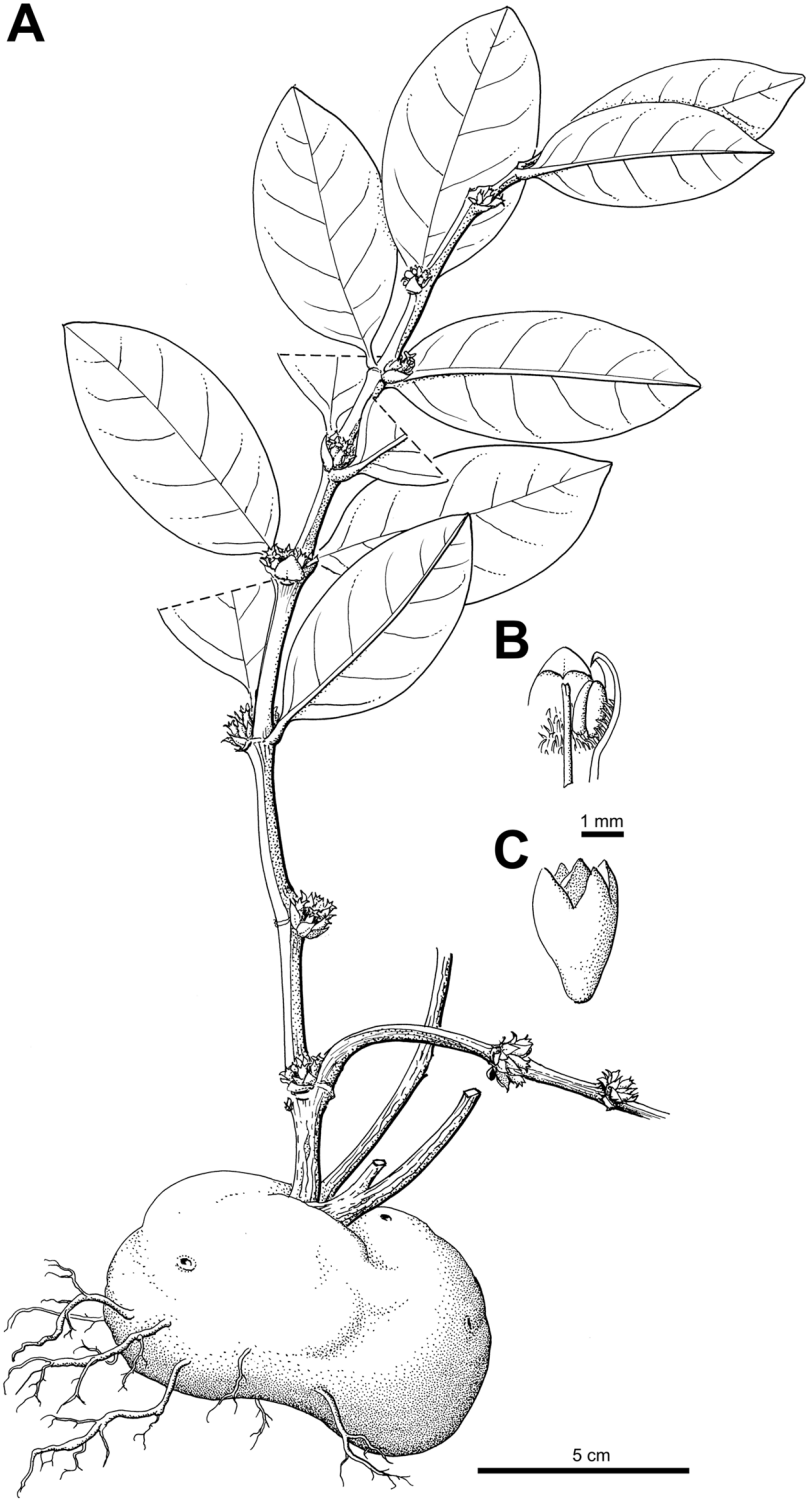
*Squamellaria vanuatuensis* Jebb & C.R.Huxley, spec. nov. (**A**). Habit showing the domatium; (**B**). Inner view of flower throat; (**C**). Hypanthium and calyx. Scale bar 5 cm for **A**; 5 mm for **B** and **C**. Drawn by Rosemary Wise from herbarium specimens: **A** = *L*. *Bernardi 13238* (G, barcode G-62446); **B, C** = Green 1274 (K, barcode K000772005).

***Squamellaria vanuatuensis*** Jebb & C.R.Huxley in Chomicki & S.S. Renner **spec. nov.** [urn:lsid:ipni.org:names: 77153598–1] (Figs 2L and 10)

#### Type

**Vanuatu, Espiritu Santo Island**: 14°57’50”S, 166°38’52”E, 600 m alt., 17 Nov. 2006, G. *McPherson*, *M*. *Tuiwawa*, *and R*. *Rigault 19437* (PVNH holotype; MO, barcode 2530839; P, barcode: P04534466, NOU barcode NOU074252, SUVA, isotypes).

#### Diagnosis

*Squamellaria vanuatuensis* differs from the other species in the genus by the presence of dense triangular papery bracts around inflorescences with minute flowers (0.5 cm vs. >1.5 cm for all other *Squamellaria* species; Fig 10). It also differs in the following substitution in the nuclear ribosomal intergenic spacer region ITS (GenBank # JX155078): (*i*) a G at position 149 instead of a C for all Fijian *Squamellaria* (G shared with *S*. *kajewskii* and *S*. *guppyanum*); (*ii*) a A at position 171 instead of G or C in all other *Squamellaria*; and (*iii*) a T in position 186 instead of a A or C in all other *Squamellaria*.

#### Description

Tuber attached to tree trunks, to 40 cm across, globose to flattened (Figs 2L and 10A), tuber surface red-brown, its cavities large, entrances holes to 1 cm in diameter. Stems several, to 60 cm long and 0.2–0.5 cm of diameter, branched, of sympodial structure, all emerging from the flattened tuber apex. Internodes 1.5 to 5 cm. Leaves arranged in a decussate phyllotaxis, lamina ovate; 4 x 2.2 to 5.5 x 3.3 cm; apex acute; base rounded; succulent; pale green. Venation dark; petiole 2 cm. Stipules to 0.15 cm, rounded, papery, caducous. Inflorescence 1–3 per node, sessile, covered by papery, triangular bracts to 1 cm in length, forming a mass 1.5 cm across. Flowers minute, bisexual, homostylous, calyx dentate, 3 mm long with teeth to 1 mm (Fig 10C), corolla 2.5 mm overall, with dense hairs at the opening (Fig 10B). Anthers ca. 1.2 mm long, basifixed, adnate to corolla, valvate, and with introrse dehiscence. Pollen 3-colpate, 57.5 μm across; reticulation medium, 1–2 μm. Ovary inferior, stigma bifid. Fruit and pyrenes unknown.

#### Distribution and ecology

*Squamellaria vanuatuensis* is known from rainforests on five islands of the Vanuatu archipelago: Erromango island, close to Nouankao camp, Efate island, Summit of Mt. Macdonald (647 m alt.), Undine Bay, and Maewo island, Saritamita. Pentecost island, close to Enkul village, Espiritu Santo Island, West coast of Cumberland peninsula, above the village of Penarou. Tuber inhabited by unspecialized ants of various genera (M.P.H. Jebb, pers. comm. to G.C., Feb. 2015).

#### Etymology and common name

Named for its geographic distribution on Vanuatu. The epithet was first used by M. Jebb and C. R. Huxley in December 1991 on annotation labels attached to *P*.*S*. *Green 1274* (K, P).

#### Conservation status

The species is known from five islands in the Vanuatu island group and thus have a small range. Although the lack of data prevents us from assigning an IUCN status to this species, we suspect, based on criteria B (extent of occurrence) and C (population size and decline) [34], that it may be endangered.

#### Specimens examined

VANUATU. **Erromango Island**: *L*. *Bernardi 13238* (G barcode G00405545); 18°54’0”S, 169°10’60”E, Erromanga, 5 Aug. 1971, *P*. *S*. *Green 1274* (K, P). **Efate Island**: Summit of Mt. Macdonald, Undine Bay, *Morrison s*.*n*. (K). **Maewo Island**: Saritamita, 23 April 1986, *Bourdy 532* (K, P). **Pentecost Island**: Near Enkul village, 500 m alt., 27 Sep. 1984, *P*. *Cabalion 2528* (K, P).

### Key to the species of *Squamellaria*

1. Species not occurring in Fiji………………………………………………………………………….2
2. Endemic to Vanuatu; inflorescence covered by triangular papery bracts (Figs 2L and 10A)………….…………………………………………………. ………………………*S*. *vanuatuensis*
2. Endemic to the Solomon Islands, inflorescence not covered by papery bracts…………………….3
3. Lamina 5–20 cm long; peduncle 3-branched, with 4 or more fertile branch ends, corolla tube slender, at least 3 times as long as broad, tuber round to ovate (Fig 2K) …………………*S*. *guppyana*
3. Lamina 2–4 cm long; peduncle 2-branched, with 2 or rarely 3 fertile branch ends; corolla tube scarcely longer than broad, tuber boat-shaped (Fig 2J)……………………………………*S*. *kajewskii*
1. Species occurring in Fiji…………………………………………………………………………….4
4. Tuber entrance holes of 0.5–5 cm, arranged irregularly and concentrated at the basal part of the tuber, leaves 2–12 cm long, flowers with thin corolla tubes (2–3 mm), inhabited by various ant species, but not *Philidris nagasau*………………………………………………………………………………5
5. Herbarium and living material of the following three species cannot be securely distinguished morphologically; endemic to the South East Fiji islands Viti Levu, Ovalau.………………*S*. *tenuiflora*
5. Endemic to the North West Fiji islands Vanua Levu and Taveuni…………………………………………6
6. Vanua Levu…………………………………………………………………………….*S*. *wilkinsonii*
6. Taveuni……………………………………………………………………………………*S*. *jebbiana*
4. Tuber entrance holes <3 mm, in circles around the tuber, leaves 3–8.5 cm, flowers with large (4–6 mm) or thin (2–3 mm) corolla tubes, always inhabited by the ant species *Philidris nagasau*………………….………………………………………………………….………………….7
7. Flower calyx 3 mm wide, corolla tubes 2.5–3.5 mm wide (Figs 3D and 4B, 4E and 4F); squamellae absent, carpels three with straight pyrenes (Fig 3J and 3M)…………………………………………………*S*. *grayi*
7. Flower calyx 5–7 mm wide, corolla tube 4–10 mm wide, squamellae present, carpels four with curved pyrenes………………………………………………………………………………………8
8. Tuber lacking hairs but with dark brown protuberances (Figs 2D and 3E); leaves oblong (Figs 2F and 4F)…………………………… ………………………………………………………… *S*. *huxleyana*
8. Tuber with hairs (*S*. *imberbis*, *S*. *wilsonii*) and/or pale protuberances (*S*. *major*, *S*. *thekii*); leaves not oblong except in *S*. *major*……………………………………………………………………………9
9. Leaves not succulent, lanceolate to rhomboid, 1–2 mm thick ………………………………………10
10. Domatium globose, with hairs; species endemic to Vanua Levu (Fig 2E)…………. *S*. *imberbis*
10. Domatium flattened, with pronounced bilateral symmetry (Fig 2B), with hairs, species endemic to Taveuni……………….……………………………………………………………………*S*. *wilsonii*
9. Leaves succulent, cordate to ovate-rhomboid or ovate to oblong-elliptic, 3–4 mm thick…………11
11. Leaves ovate to oblong-elliptic, 8–16 cm long, stems solitary around the domatium (Fig 2C)……….…………………………………………………………………………………… *S*. *major*
11. Leaves cordate to ovate-rhomboid, 4–6 cm long, stems in clusters around the tuber (Fig 2A)………….…………………………………………………………………………………………………………*S*. *thekii*

### New combinations

*Squamellaria guppyana* (Becc.) Chomicki, **comb. nov.** [urn:lsid:ipni.org:names:77153477–1] Basionym: *Hydnophytum guppyanum* Becc., Malesia 2: 133, pl. 40. 1885. **Type**: Solomon Islands, Shortland islands, May 1884, *H*.*B*. *Guppy s*.*n*. (FI holotype, barcode: FI008898).

Note: Kew has no duplicate of this collection, but instead *H*.*B*. *Guppy 140* (image barcode K000772006), which appears to represent the same collection.

*Squamellaria kajewskii* (Merr. & L.M.Perry) Chomicki, **comb. nov.** [urn:lsid:ipni.org:names: 77153478–1] Basionym: *Hydnophytum kajewskii* Merr. & L.M.Perry, J. Arnold Arbor. 26: 25. 1945. **Type**: Solomon Islands, Bougainville, April 1930, *S*.*F*. *Kajewski 1716* (A holotype, barcode: A00096843; BM, barcode: BM001040409, BO, BRI, barcode BRI-AQ0570119, G, barcode G00436269, P, barcode P04957009 isotypes).

*Squamellaria tenuiflora* (Becc.) Chomicki, **comb. nov.** [urn:lsid:ipni.org:names:77153479–1] Basionym: *Hydnophytum tenuiflorum* Becc., Malesia 2: 169, pl. 43, 1–14. 1885. **Syntypes**: Fiji, Viti Levu, Dec. 1864, *E*.*O*. *Graeffe 1573* (K, barcode: K000761993, lectotype designated by A.C.Smith, Fl. Vitiensis Nova 4: 244. 1988), Ovalau, *E*.*O*. *Graeffe 1555* (K, paratype, barcode: K000761992).

Gray and Beccari described three further species that may be synonyms of *S*. *tenuiflora*, but without DNA sequences from their type specimens it is not currently possible to decide the matter. They are *H*. *longiflorum* A.Gray (Proc. Am. Acad. 4: 42. 1858) based on US Expl. Exp. 62267 (US Catalog No. 62267, barcode: 00036508) from Ovalau; *H*. *grandiflorum* Becc. (Malesia 2: 126. 1884, Malesia 2: 171, pl. 44: 13–25. 1885) based on *E*.*O*. *Graeffe s*.*n*. (K barcode 000762000) from ‘Fiji, Ovalau and Viti Levu, Dec. 1864’; and *H*. *horneanum* Becc. (Malesia 2: 125. 1884, Malesia 2: 168, pl. 43:15–25, 1885) based on *J*. *Horne 282* (K barcode: K000761999) from Fiji.

*Squamellaria wilkinsonii* (Horne ex Baker) Chomicki, **comb. nov.** [urn:lsid:ipni.org:names: 77153480–1] Basionym: *Hydnophytum wilkinsonii* Horne ex Baker, J. Linn. Soc., Bot. 20: 365. 1883 [1884 publ. 1883]. **Type**: Fiji, Vanua Levu, 1877–78, *J*. *Horne 1077* (K holotype, barcode: K000761990; K000761991, isotype).

*Squamellaria wilkinsonii* from Vanua Levu and the preceding species, S. *tenuiflora* from Viti Levu, are morphologically extremely similar.

## Discussion

### Traits and taxonomy of the Fijian Hydnophytinae (Psychotrieae, Rubiaceae)

The genus *Squamellaria* now comprises 12 species, four of them described here, four transferred from *Hydnophytum*, and one resurrected. The resurrected species is *S*. *wilsonii*, which was synonymized under *S*. *imberbis* by Jebb [6] but is distinct in tuber shape and DNA sequences (Fig 2B and 2M). The entire subtribe Hydnophytinae (*Anthorrhiza*, *Hydnophytum*, *Myrmecodia*, *Myrmephytum*, *Squamellaria*) is embedded in *Psychotria* (Fig 1), and Razafimandimbison et al. [36] have therefore transferred the type species of each of these genera into *Psychotria*, but without transferring the remaining names. For three reasons, we decided to describe our new species in *Squamellaria* instead of in *Psychotria*. Firstly, some 4000 names are available in *Psychotria*, and any transfers into that genus are therefore best left to *Psychotria* specialists. Secondly, Matthew Jebb (National Botanic Gardens, Glasnevin, Dublin) is working on a taxonomic revision of *Hydnophytum*. Thirdly, species and gene sampling densities in the Pacific clade of *Psychotria* are still sparse [29, 36], and two more broadly defined genera could be maintained separate from *Psychotria*, namely our expanded *Squamellaria* and the Australasian clade as an expanded *Hydnophytum* (Fig 1). Nevertheless, we selected the epithets of our four new species so as not to require replacement names should these species be transferred into *Psychotria* in the future. The genus *Squamellaria* can be defined by the combination of 4-merous sepal and petal whorls together with solitary inflorescences (except in *S*. *vanuatuensis* where this trait is variable) and distribution in the Pacific (Fiji, Vanuatu, Solomons).

The scales at the inner base of the flower tube used by Beccari [14] to set apart *Squamellaria* from *Hydnophytum* can no longer be used as the defining morphological trait of the genus, since they only arose within the genus, for example in *S*. *grayi* (species followed with a “S” in Fig 2). *Squamellaria grayi* is distinctive by the slight succulence and curved shape of its leaves (Figs 3A and 4A). The isotope *δ*^*13*^*C* ratio for CAM versus C3 photosynthesis revealed that these leaves carry out standard C3 photosynthesis, as do *S*. *jebbiana*, *S*. *imberbis*, and *S*. *wilsonii*, while *S*. *huxleyana*, *S*. *major*, and *S*. *thekii* have CAM photosynthesis or intermediate C3/CAM photosynthesis.

### Biogeographic history of *Squamellaria*

During the Oligocene, some 30 million years ago, Fiji, Vanuatu, the Solomon Islands, and the Bismarck Archipelago were part of a volcanic arc, the Vitiaz arc, with active volcanism that continued to build land [37]. By about 12 Ma, the Solomons had rifted from the Vanuatu-Fijian arc, and the latter two regions then became separated at about 7 Ma [32, 38]. The inferred ancestral area for *Squamellaria* in Fiji and Vanuatu is thus consistent with the Vitiaz arc (Fig 1). The colonization of Vanuatu by the ancestor of *S*. *vanuatuensis* could have occurred from Fiji, and the common ancestor of *S*. *kajewskii* and *S*. *guppyana* later reached the Solomon Islands (Fig 1). The six *Squamellaria* species (marked in red in Fig 2) that live in an obligate symbiosis with a single ant species (*Philidris nagasau*) are restricted to Taveuni and Vanua Levu together with their symbiont, two islands separated by only 6.5 km, implying that they arrived as epiphytes on floating tree trunks, with their domatia occupied by their own coevolved ants. By contrast, the *Squamellaria* species occupied by facultative ant symbionts are widespread on the archipelago. A biogeographic analysis of Neotropical *Pseudomyrmex* ants and their plant hosts showed that interacting ant and plant clades shared the same ancestral areas and that dispersal events outside of the partner ranges were rare [39].

One of our new species, *S*. *jebbiana* from Taveuni, is sister to the other Fijian *Squamellaria* (Figs 1 and 9) from which it appears to have diverged 3.7 ± 1.5 Ma (Fig 1). This is puzzling because Taveuni is supposed to have emerged only some 0.8 Ma ago [40, 41]. Perhaps the species arrived from an older island, such as nearby Vanua Levu (~4 Ma old; [41]) or Viti Levu (~28 Ma; [41]), followed by local extinction on these islands. Alternatively, the uncertainty of molecular clock dating, especially of young nodes for which error ranges cannot be calculated because of too few substitutions, may explain the age discrepancy between the island age and the inferred species divergence time.

## Conclusion

Our four new species, four new combinations, and the resurrected *S*. *wilsonii* bring the number of ant-plant species worldwide to 685 [1]. The discovery of five new myrmecophytes on tiny islands of the Fiji Archipelago suggests that a modelling-based estimate of probably over 1,100 myrmecophyte species worldwide may well be realistic [1]. That new species were discovered on a tourist walk also illustrates how much botanical collecting remains to be done on Fiji.

## Supporting Information

S1 Table Plant material included in this study, with species authors, vouchers and their geographic origin, GenBank accession numbers for all sequences. Herbarium acronyms follow the *Index Herbariorum* (http://sciweb.nybg.org/science2/IndexHerbariorum.asp).
(DOCX)

## References

[1] Chomicki G, Renner SS. Phylogenetics and molecular-clock dating reveal the repeated evolution of ant-plants after the late Miocene in Africa and the early Miocene in Australasia and the Neotropics. New Phytol. 2015; 207: 411–424. 10.1111/nph.13271 25616013

[2] Huxley CR, Jebb MHP. The tuberous epiphytes of the Rubiaceae 1: A new subtribe- the Hydnophytinae. Blumea. 1991; 36: 1–20.

[3] Huxley CR, Jebb MHP. The tuberous epiphytes of the Rubiaceae 5: A revision of *Myrmecodia*. Blumea 1993; 37: 271–334.

[4] Huxley CR, Jebb MHP. The tuberous epiphytes of the Rubiaceae: 3. A revision of *Myrmephytum* to include *Myrmedoma*. Blumea 1991; 36: 43–52.

[5] Huxley CR, Jebb MHP The tuberous epiphytes of the Rubiaceae 2: the new genus *Anthorrhiza*. Blumea. 1991; 36: 21–41.

[6] Jebb MHP. The tuberous epiphytes of the Rubiaceae: 4. A revision of *Squamellaria*. Blumea 1991; 36: 53–61.

[7] Treub MM. Sur le *Myrmecodia echinata* Gaudich. Ann Jard Bot Buitenz. 1883; 3: 129–59.

[8] Treub MM. Nouvelles recherches sur le *Myrmecodia* de Java. Ann Jard Bot Buitenz. 1888; 7: 191.

[9] Huxley CR. The ant-plants *Myrmecodia* and *Hydnophytum* (Rubiaceae), and the relationships between their morphology, ant occupants, physiology and ecology. New Phytol. 1978; 80: 231–268.

[10] Miehe H. Ueber die javanische *Myrmecodia* und die Beziehung zu ihren Ameisen. Biol Zbl. 1991; 31: 73

[11] Heim DR. The biologic relations between plants and ants Smithsonian Report for 1896. 1988; 411–455. Government Printing Office, Washington.

[12] Janzen DH. Epiphytic myrmecophytes in Sarawak: mutualism through the feeding of plants by ants. *Biotropica*. 1974; 6: 237–259.

[13] Barosi F. Odoardo Beccari. I viaggi e il contributo scientifico. Geostorie. 2010; 18: 7–85.

[14] Beccari O. Piante ospitatrici, ossia piante formicarie della Malesia e della Papuasia. 1884–1886; *Malesia (Genoa)* vol. II, fasc. 1–2 (1884), fasc. 3 (1885), fasc. 2 (1886).

[15] Horne J. A year in Fiji, or an Inquiry Into the Botanical, Agricultural, and Economical Resources of the Colony. 1881; London: Eyre GE & Spottiswoode W.

[16] Baker JG. Recent additions to our knowledge of the flora of Fiji. J Linn Soc, Bot. 1884 [published in 1883] 20: 358–373.

[17] Wagner WL, Lorence DH. Albert Charles Smith (1906–1999): a monumental botanist. Allertonia 2001; 8: 329–339.

[18] Lin H-L. Colonial uneven development, Fijian Vanua, and modern ecotourism in Taveuni, Fiji. Pacific Asia Inquiry. 2012; 3: 41–57.

[19] Smith AC. Studies of the Pacific Islands plants XVIII. New and noteworthy plants from Fiji. Contr US Natl Herb. 1967; 37: 67–107.

[20] Chomicki G, Renner SS. Watermelon origin solved with molecular phylogenetics including Linnaean material: Another example of museomics. New Phytol. 2015; 205: 526–532. 10.1111/nph.13163 25358433

[21] Katoh K, Standley DM. MAFFT multiple sequence alignment software version 7: improvements in performance and usability. Mol Biol Evol. 2013, 30: 772–780. 10.1093/molbev/mst010 23329690 PMC3603318

[22] Maddison WP, Maddison DR. Mesquite: a modular system for evolutionary analysis. Version 2.75. 2011. *URL* http://mesquiteproject *org*.

[23] Stamatakis A. RAxML version 8: a tool for phylogenetic analysis and post-analysis of large phylogenies. Bioinformatics. 2014; 30: 1312–1313. 10.1093/bioinformatics/btu033 24451623 PMC3998144

[24] Ronquist F, Teslenko M, van der Mark P, Ayres DL, Darling A, Höhna S et al MrBayes 3.2: efficient Bayesian phylogenetic inference and model choice across a large model space. Syst Biol. 2012; 61: 539–542. 10.1093/sysbio/sys029 22357727 PMC3329765

[25] Darriba D, Taboada GL, Doallo R, Posada D. jModelTest 2: more models, new heuristics and parallel computing. Nat Methods. 2012; 9: 772. 10.1038/nmeth.2109 PMC4594756 22847109

[26] Bouckaert R, Heled J, Kühnert D, Vaughan T, Wu CH, Xie D et al BEAST 2: a software platform for Bayesian evolutionary analysis. PLoS Comp Biol. 2014; 104, e1003537. 10.1371/journal.pcbi.1003537 PMC3985171 24722319

[27] Rambaut A, Drummond AJ. Tracer—MCMC trace analysis tool version v1.5. 2007; URL http://beast.bio.ed.ac.uk.

[28] Rambaut A. FigTree v. 1.4.0. 2012. http://tree.bio.ed.ac.uk/software/figtre.

[29] Barrabé L, Maggia L, Pillon Y, Rigault F, Mouly A, Davis AP, et al New Caledonian lineages of *Psychotria* (Rubiaceae) reveal different evolutionary histories and the largest documented plant radiation for the archipelago. Molecular Phylogenet Evol. 2014; 71: 15–35. 10.1016/j.ympev.2013.10.020 24211193

[30] Matzke NJ. Founder-event speciation in BioGeoBEARS package dramatically improves likelihoods and alters parameter inference in dispersal–extinction–cladogenesis DEC analyses. Front Biogeog. 2012; 4: 210.

[31] Matzke NJ. Model selection in historical biogeography reveals that founder-event speciation is a crucial process in island clades. Syst Biol. 2014; 63: 951–970. 10.1093/sysbio/syu056 25123369

[32] Hall R. Cenozoic geological and plate tectonic evolution of SE Asia and the SW Pacific: computer-based reconstructions, models, and animations. J Asian Earth Sci. 2002; 20: 353–431.

[33] Gray A. Myrmecodia imberbis. Proc Am Acad Arts Sci. 1858; 4: 31–78.

[34] IUCN Species Survival Commission. *IUCN Red List Categories and Criteria*. 2001 IUCN.

[35] Jebb M. Cavity structure and function in the tuberous Rubiaceae 1991; Huxley CR, Cutler D, F ed (s). Ant-plant interactions. Oxford Univ. Press: Oxford, p.p. 374–89.

[36] Razafimandimbison SG, Taylor CM, Wikström N, Pailler T, Khodabandeh A, Bremer B. Phylogeny and generic limits in the sister tribes Psychotrieae and Palicoureeae (Rubiaceae): Evolution of schizocarps in *Psychotria* and origins of bacterial leaf nodules of the Malagasy species. Am J Bot. 2014; 101: 1102–1126. 25049266 10.3732/ajb.1400076

[37] Ewart FT. Geological history of the Fiji–Tonga–Samoan Region of the S.W. Pacific, and some palaeogeographic and biogeographic implications 1988; In: Lyneborg L. (Ed.). The Cicadas of the Fiji, Samoa and Tonga Islands, Their Taxonomy and Biogeography. EJ Brill/Scandinavian Science Press, Leiden, Netherlands.

[38] Taylor GK, Gascoyne J, Colley H. Rapid rotation of Fiji; paleomagnetic evidence and tectonic implications. J Geophys Res, B, Solid Earth Planets. 2000; 105: 5771–5781.

[39] Chomicki G, Ward PS, Renner SS. Macroevolutionary assembly of ant/plant symbioses: *Pseudomyrmex* ants and their ant-housing plants in the Neotropics. Proc R Soc B. 2015; 282: 20152200 10.1098/rspb.2015.2200 26582029 PMC4685824

[40] Rodda P, Kroenke L. Fiji: a fragmented arc, p. 87–110. In: Cenozoic Tectonic Development of the Southwest Pacific. Vol. Technical Bulletin. 1984; No. 6 Kroenke L. (ed.). U.N. ESCAP, CCOP/SOPAC.

[41] Rodda P. Geology of Fiji. South Pacific Applied Geoscience Commission (SOPAC). Technical Bulletin. 1994; 8: 131–151.

