# Supplementary material for: Evolutionary Relationships and Biogeography of the Ant-Epiphytic Genus Squamellaria (Rubiaceae: Psychotrieae) and Their Taxonomic Implications
Source: PLoS One. 2016 Mar 30;11(3):e0151317. doi: 10.1371/journal.pone.0151317 (PMC4814088; doi:10.1371/journal.pone.0151317)
Supplement: S1 Table — Herbarium acronyms follow the Index Herbariorum (http://sciweb.nybg.org/science2/IndexHerbariorum.asp). (DOCX) [file pone.0151317.s001.docx]

**S1 Table**. Plant material included in this study, with species authors, vouchers and their geographic origin, GenBank accession numbers for all sequences. Herbarium acronyms follow the *Index Herbariorum* (<http://sciweb.nybg.org/science2/IndexHerbariorum.asp>).

| Taxon | Voucher | Geographic origin | ITS | ETS | 18S | trnL-trnF region | ndhF | trnS-trnG | rps16 | rps12-rpl20 |
| --- | --- | --- | --- | --- | --- | --- | --- | --- | --- | --- |
| *Amaracarpus* *muscifer* A.C.Sm. | L. Barrabe & M. Tuiwawa 1109 (NOU) | Fiji | KF675907 | KF675790 | - | - | KF675995 | - | KF676083 | - |
| *Amaracarpus* *nematopodus* (F.Muell.) P.I.Forst. | L. Barrabe et al. 1030 (NOU) | Australia | JX155060 | KF675791 | - | - | JX155105 | - | JX155152 | - |
| *Anthorrhiza caerulea* Huxley & Jebb | M.P.H. Jebb 358 (FHO) | Papua New Guinea | KU586349 | KU586368 | - | - | - | - | - | - |
| *Anthorrhiza echinella* | G. Chomicki 83 (M) | Cultivated Oxf. Bot Gard., origin New Guinea | KU586350 | KU586369 | - | - | - | - | - | - |
| *Hedstromia* *latifolia* A.C.Sm. | L. Barrabe et al 1090 (NOU) | Fiji | KF675911 | KF675795 | - | - | KF675999 | - | KF676087 | - |
| *Hydnophytum formicarum* Jack | G. Chomicki 87 (M) | Cultivated, origin Malaysian region | KU586346 | KU586365 | - | - | KU586397 | - | - | - |
| *Myrmecodia beccarii* Hook f. | G. Chomicki 99 (M) | Cultivated, origin Australia | KU586347 | KU586366 | - | - | KU586398 | - | - | - |
| *Myrmecodia salomonensis* Becc. | C. R. Huxley and L. M. Turton 3442 (FHO) | Solomons | KU586351 | KU586370 | - | - | - | - | - | - |
| *Myrmecodia* *dahlii* K.Schum. | J.I. Menzies 5947 (FHO) | Papua New Guinea | KU586348 | KU586367 | - | - | KU586399 | - | - | - |
| *Myrmephytum arfakianum* (Becc.) Huxley & Jebb | G. Chomicki 116 (M) | Cultivated, origin Papua | KU586352 | KU586371 | - | - | KU586400 | - | - | - |
| *Myrmephytum beccarii* Elmer | G. Chomicki 118 (M) | Cultivated, origin Philippines | KU586353 | KU586354 | - | - | KU586401 | - | - | - |
| *Psychotria* *comptonii* S.Moore | L. Barrabe & Rigault 1014 (NOU) | New Caledonia | KF675927 | KF675823 | - | - | KF676015 | - | KF676104 | - |
| *Psychotria* *dallachiana* Benth. | L. Barrabe & Rigault 1048 (NOU) | Australia | KF675928 | KF675824 | - | - | KF676016 | - | KF676169 | - |
| *Psychotria* *declieuxioides* S.Moore | L. Barrabe & Nigote 937 (NOU) | New Caledonia | KF675932 | KF675828 | - | - | KF676020 | - | KF676107 | - |
| *Psychotria* *faguetii* (Baill.) Schltr. | L. Barrabe et al. 820 (NOU) | New Caledonia | KF675934 | KF675831 | - | - | KF676023 | - | - | - |
| *Psychotria* *fitzalanii* Benth. | L. Barrabe & Rigault 1057 (NOU) | Australia | KF675935 | KF675832 | - | - | KF676024 | - | KF676110 | - |
| *Psychotria* *goniocarpa* (Baill.) Guillaumin | L. Barrabe 586 (NOU) | New Caledonia | KF675940 | KF675838 | - | - | KF676029 | - | KF676115 | - |
| [*Psychotria* *hawaiiensis* (A.Gray) Fosberg](http://www.theplantlist.org/tpl1.1/record/kew-168216) | Y. Pillon 1425 (NOU) | Hawaii | KF675941 | KF675840 | - | - | KF676030 | - | KF676116 | - |
| *Psychotria* *hivaoana* Fosberg | Meyer 3071 (PAP) | French Polynesia | KF675942 | KF675841 | - | - | KF676031 | - | KF676117 | - |
| [*Psychotria* *insularum* A.Gray](http://www.theplantlist.org/tpl1.1/record/kew-168378) | Y. Pillon 909 (NOU) | Wallis & Futuna | KF675943 | KF675842 | - | - | KF676032 | - | KF676118 | - |
| *Psychotria* *iteophylla* Stapf | Axelius 303 (S) | Borneo | - | - | - | - | - | - | AF410726 | - |
| [*Psychotria* *loniceroides* Sieber ex DC.](http://www.theplantlist.org/tpl1.1/record/kew-168660) | L. Barrabe & Rigault 1042 (NOU) | Australia | KF675945 | KF675846 | - | - | KF676033 | - | KF676120 | - |
| *Psychotria* *lorentzii* Valeton | Puradyatmika 10460 (K) | Papua New Guinea | KF675946 | KF675847 | - | - | KF676034 | - | KF676121 | - |
| *Psychotria* *micralabastra* (Lauterb. & K.Schum.) Valeton | Takeuchi 16163 (K) | Papua New Guinea | KF675949 | KF675851 | - | - | KF676036 | - | KF676124 | - |
| *Psychotria* *micrococca* (Lauterb. & K.Schum.) Valeton | Drozd & Molem s.n. (PSF) | Papua New Guinea | KF675951 | KF675853 | - | - | KF676038 | - | KF676126 | - |
| *Psychotria* *microglossa* (Baill.) Baill. ex Guillaumin | L. Barrabe 585 (NOU) | New Caledonia | KF675950 | KF675852 | - | - | KF676037 | - | KF676125 | - |
| *Psychotria* *monanthos* (Baill.) Schltr. | Y. Pillon 1370 (NOU) | New Caledonia | KF675953 | KF675855 | - | - | KF676040 | - | KF676128 | - |
| [*Psychotria* *poissoniana* (Baill.) Guillaumin](http://www.theplantlist.org/tpl1.1/record/kew-274312) | J. Munzinger 5156 (NOU) | New Caledonia | KF675958 | KF675861 | - | - | KF676045 | - | KF676133 | - |
| *Psychotria* *pritchardii* Seem. | L. Barrabe et al 1124 (NOU) | Fiji | KF675992 | KF675903 | - | - | KF676078 | - | KF676165 | - |
| *Psychotria* *raivavaensis* Fosberg | Meyer 3088 (PAP) | French Polynesia | KF675960 | - | - | - | KF676047 | - | KF676135 | - |
| *Psychotria* *submontana* Domin | L. Barrabe et al. 1044 (NOU) | Australia | KF675988 | KF675899 | - | - | - | - | KF676168 | - |
| *Psychotria* *temehaniensis* J.W.Moore | Mouly 403 (P) | French Polynesia | KF675989 | KF675900 | - | - | KF676075 | - | KF676162 | - |
| *Psychotria* *trisulcata* (Baill.) Guillaumin | L. Barrabe et al. 902 (NOU) | New Caledonia | KF675990 | KF675901 | - | - | KF676076 | - | KF676163 | - |
| *Squamellaria grayi* Chomicki & Wistuba sp. nov. | G. Chomicki, J. Aroles, A. Naikatini 53 (SUVA)  HOLOTYPE | Taveuni, Bouma falls, Lavena | KU586339 | KU586358 | KU586427 | KU586376 | KU586388 | KU586406 | KU586436 | KU586417 |
| *Squamellaria grayi* Chomicki & Wistuba sp. nov. | G. Chomicki, J. Aroles, A. Naikatini 47 (M) | Vanua Levu, Waisali forest park | - | - | - | KU586372 | - | KU586402 | KU586432 | KU586413 |
| *Squamellaria guppyana* (Becc.) Chomicki, comb. nov. | G. Chomicki 123 (M) | Cultivated, origin Solomons | KU586345 | - | - | - | KU586396 | - | - | - |
| *Squamellaria huxleyana* Chomicki sp. nov. | G. Chomicki, J. Aroles, A. Naikatini 48 (SUVA)  HOLOTYPE | Fiji, Vanua Levu, road between Savusavu to Labasa. | KU586336 | KU586355 | KU586425 | KU586373 | KU586385 | KU586403 | KU586433 | KU586414 |
| *Squamellaria imberbis* (A. Gray) Becc. | G. Chomicki, J. Aroles, A. Naikatini 50 (M) | Fiji, Vanua Levu, track to vodaphone tower. | KU586337 | KU586356 | - | KU586374 | KU586386 | KU586404 | KU586434 | KU586415 |
| *Squamellaria jebbiana* Chomicki, sp. nov. | G. Chomicki, J. Aroles, A. Naikatini 74 (M) | Fiji, Taveuni, Mt Manuca area. | KU586342 | KU586361 | - | KU586379 | KU586391 | KU586408 | KU586438 | KU586419 |
| *Squamellaria kajewskii* (Merr. & L.M.Perry) Chomicki, comb. nov. | G. Chomicki 122 (M) | Cultivated, origin Solomons | KU586335 | - | - | - | KU586384 | - | - | - |
| *Squamellaria major* A.C. Sm. | G. Chomicki, J. Aroles, A. Naikatini 61 (M) | Fiji, Taveuni, road to DesVoeux peak. | KU586338 | KU586357 | KU586426 | KU586375 | KU586387 | KU586405 | KU586435 | KU586416 |
| *Squamellaria tenuiflora* (Becc.) Chomicki, comb. nov. | G. Chomicki, J. Aroles, A. Naikatini 75 (M) | Fiji, Viti Levu, Colo-i-Suva forest park. | - | - | KU586430 | KU586381 | KU586393 | KU586410 | KU586440 | KU586421 |
| *Squamellaria tenuiflora* (Becc.) Chomicki, comb. nov. | G. Chomicki, J. Aroles, A. Naikatini 78 (M) | Fiji, Viti Levu, Colo-i-Suva forest park. | KU586343 | KU586362 | KU586431 | KU586382 | KU586394 | KU586411 | - | KU586422 |
| *Squamellaria thekii* Jebb | G. Chomicki, J. Aroles, A. Naikatini 57 (M) | Fiji, Taveuni, road to DesVoeux peak. | KU586340 | KU586359 | KU586428 | KU586377 | KU586389 | KU586407 | KU586437 | KU586418 |
| *Squamellaria vanuatuensis* (Jebb & Huxley) Chomicki, comb. nov. | McPherson 19437 (P) | Vanuatu | JX155078 | - | - | - | - | - | JX155170 | - |
| *Squamellaria wilkinsonii* (Horne ex Baker) Chomicki, comb. nov. | G. Chomicki, J. Aroles, A. Naikatini 43 (M) | Fiji, Vanua Levu, Waisali forest park. | - | - | KU586429 | KU586380 | KU586392 | KU586409 | KU586439 | KU586420 |
| *Squamellaria wilkinsonii* (Horne ex Baker) Chomicki, comb. nov. | G. Chomicki, J. Aroles, A. Naikatini 49 (M) | Fiji, Vanua Levu, Waisali forest park. | - | KU586364 | - | - | - | - | - | - |
| *Squamellaria wilkinsonii* (Horne ex Baker) Chomicki, comb. nov. | G. Chomicki, J. Aroles, A. Naikatini 45 (M) | Fiji, Vanua Levu, Waisali forest park. | KU586344 | KU586363 | - | KU586383 | KU586395 | KU586412 | KU586441 | KU586423 |
| *Squamellaria wilsonii* (Horne ex Baker) Becc. | G. Chomicki, J. Aroles, A. Naikatini 67 (M) | Fiji, Taveuni, road to DesVoeux peak. | KU586341 | KU586360 | - | KU586378 | KU586390 | - | - | KU586424 |
